# Tracking and classifying Amazon fire events in near real time

**DOI:** 10.1126/sciadv.abd2713

**Published:** 2022-07-29

**Authors:** Niels Andela, Douglas C. Morton, Wilfrid Schroeder, Yang Chen, Paulo M. Brando, James T. Randerson

**Affiliations:** ^1^ School of Earth and Environmental Sciences, Cardiff University, Cardiff, UK.; ^2^ BeZero Carbon, London, UK.; ^3^ Biospheric Sciences Laboratory, NASA Goddard Space Flight Center, Greenbelt, MD 20771, USA.; ^4^ NOAA NESDIS, College Park, MD 20740, USA.; ^5^ Department of Earth System Science, University of California, Irvine, CA 92697, USA.; ^6^ Instituto de Pesquisa Ambiental da Amazônia (IPAM), SHIN, CA-5, Brasilia, DF 7500, Brazil.; ^7^ Woods Hole Research Center, 149 Woods Hole Rd., Falmouth, MA 02540, USA.

## Abstract

Exceptional fire activity in 2019 sparked concern about Amazon forest conservation. However, the inability to rapidly separate satellite fire detections by fire type hampered fire suppression and assessment of ecosystem and air quality impacts. Here, we describe the development of a near–real-time approach for tracking contributions from deforestation, forest, agricultural, and savanna fires to burned area and emissions and apply the approach to the 2019 fire season in South America. Across the southern Amazon, 19,700 deforestation fire events accounted for 39% of all satellite active fire detections and the majority of fire carbon emissions (63%; 69 Tg C). Multiday fires accounted for 81% of burned area and 92% of carbon emissions from the Amazon, with many forest fires burning uncontrolled for weeks. Most fire detections from deforestation fires were correctly identified within 2 days (67%), highlighting the potential to improve situational awareness and management outcomes during fire emergencies.

## Teaser

Near–real-time tracking of individual fires can distinguish fire types to support rapid responses to Amazon fire emergencies.

## INTRODUCTION

The Amazon is the largest tropical forest on Earth and critical for climate regulation (*1*), carbon sequestration (*2*), and biodiversity conservation (*3*). Over the past six decades, demand for agricultural products has spurred fire-driven deforestation to convert Amazon forest into extensive pastures and croplands (*4*–*6*). Nevertheless, satellite monitoring capabilities (*7*), governance (*8*), and industry actions to exclude deforestation from commodity supply chains (*9*) resulted in a strong and sustained decline in deforestation rates and associated fire activity in the Brazilian Amazon (*6*), the most active Amazon deforestation frontier (*10*). The recent reversal in these deforestation trends, followed by an uptick in regional fire activity in 2019 (*11*), therefore ignited a global discussion about what type of fires were burning.

**Fig. 1.**
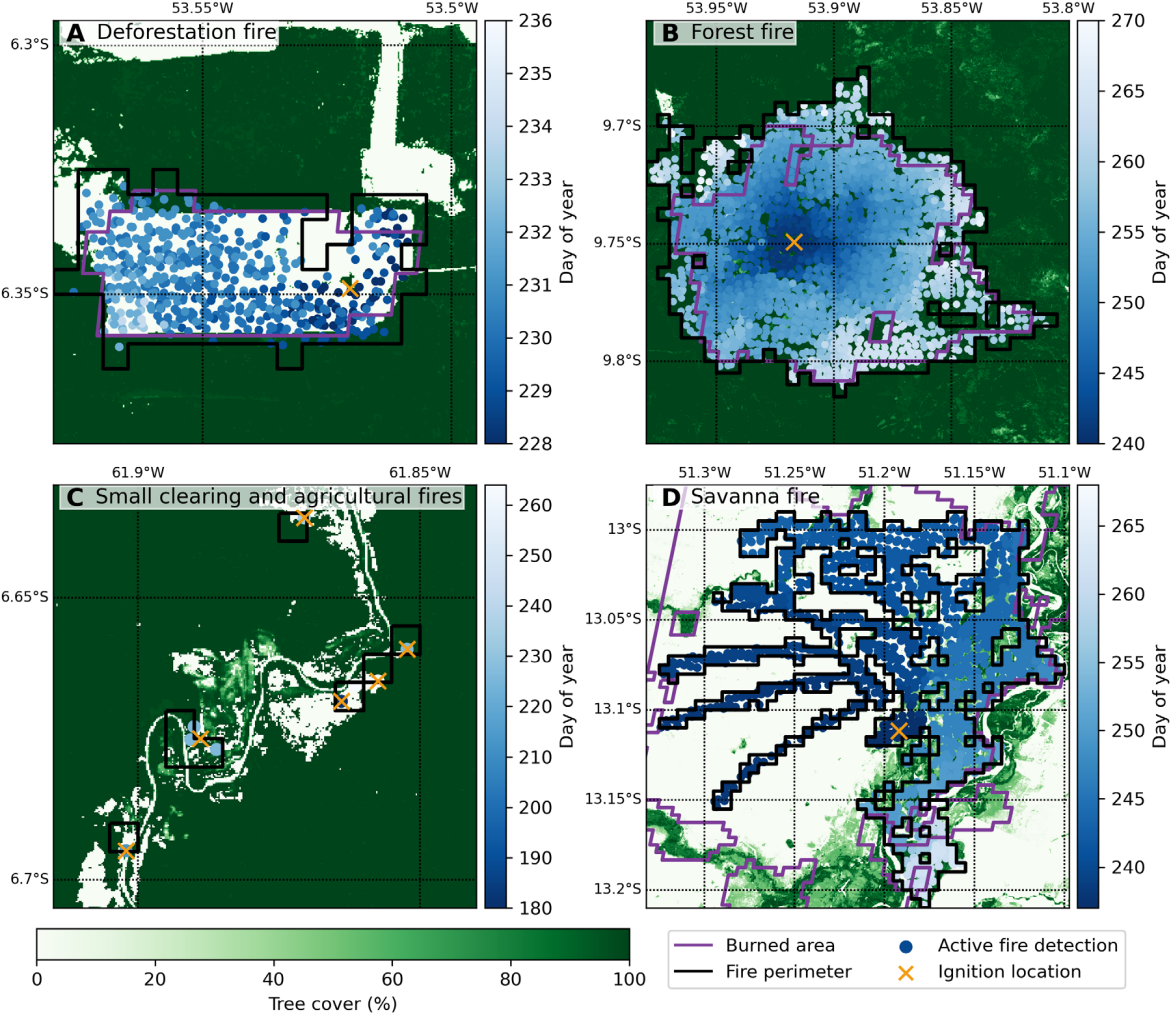
The four dominant fire types in the Amazon region have distinct patterns of fire behavior. (**A**) Deforestation fire, (**B**) understory forest fire, (**C**) small clearing and agricultural management fires, and (**D**) savanna fire spreading into gallery forests with higher fractional tree cover (*10*). VIIRS active fire detections were clustered and classified by fire type using metrics of fire behavior and land cover information (*25*). Active fires (circles) are colored by day of year. Black lines indicate the fire perimeters from clustering VIIRS active fire detections into individual events, purple lines show the extent of satellite burned-area data (*29*), and orange crosses mark the estimated ignition locations. Each 0.05° grid box is approximately 5.5 km by 5.5 km.

Fires in the Amazon basin, nearly all of which are started by humans, can be classified into four broad categories on the basis of land cover and land management (Fig. 1). First, deforestation fires include initial burning following forest clearing and subsequent fires to burn piled woody debris, stumps, and roots. These fires are undertaken to prepare land for use as pasture or cropland (*12*). On the basis of higher fuel loads, repeated burning, and long-term conversion to nonforest land uses, deforestation fires have a large impact on net carbon emissions from Amazon forests (*13*). Second, forest fires occur when deforestation or agricultural fires intentionally or accidentally escape into the understory of neighboring forests. This class of fire is particularly damaging; fire-induced tree mortality often exceeds 50% of aboveground biomass (*14*), as Amazon forest trees have thin bark and other traits that make them susceptible to even low-intensity forest fires (*15*, *16*). Forest fires are therefore a leading cause of regional forest degradation (*17*, *18*), especially during drought years (*19*). Third, fires are a common tool for agricultural management, including land clearing in rotational slash-and-burn cropland systems, preparation for planting, and pasture maintenance (*12*), resulting in an abundance of small or short-duration fires across the region that do not spread over multiple days (*20*). Fourth, the Amazon biome also encompasses savanna and grassland ecosystems, where fire plays a key role in regulating ecosystem function and the evolution of different plant and animal species (*21*). Large grassland fires may also occur in pasture lands where intentional or accidental ignitions burn for many days across ranches and farms along the agricultural frontier. The contributions from these four fire types to regional fire activity remain poorly understood (*11*), partly because of the scale mismatch between the fragmentation of human-dominated landscapes and the coarse resolution of satellite fire detections (*22*, *23*), and the challenges associated with detecting understory forest fires (*24*). Separating these fire types is critical for constraining fire carbon emissions (*13*), quantifying fire effects on forested ecosystems (*14*, *15*), and targeting suppression efforts to mitigate threats to forest conservation and regional air quality from fire.

Here, we fill this critical data gap (*11*) on the relative contribution from different fire types to total Amazon fire activity using a new approach to track and classify individual fires in near real time. As a demonstration, we apply the approach to the 2019 fire season in the southern Amazon, a year that sparked controversy because long-distance transport of smoke from biomass burning in the Amazon and surrounding biomes degraded air quality in remote cities, including São Paulo. Our approach clusters satellite fire detections into individual fires using the Global Fire Atlas (*25*) methodology (fig. S1), based on the finer 375-m spatial resolution and better sensitivity of the Visible Infrared Imaging Radiometer Suite (VIIRS) instruments (*26*) onboard the Suomi-NPP and NOAA-20 satellites. Starting in April 2019, the two VIIRS instruments provide daily, near-nadir observations needed to detect both high- and low-intensity fires (Fig. 1). Our method combines multiday metrics of fire behavior and land cover information with an expert-guided classification approach to classify individual fire events by fire type (figs. S2 to S5). We compare fire events derived from VIIRS detections to independent maps of deforestation (*6*), fire event data from the Monitoring of the Andean Amazon Project (MAAP) (*27*), and pre- and postfire Sentinel-2 imagery for a stratified random subset of fire events to evaluate the accuracy of the classification both in near real time and at the end of the year (figs. S6 to S10 and table S2). To estimate carbon emissions associated with each fire event (figs. S11 to S15), we rely on the Moderate Resolution Imaging Spectroradiometer (MODIS) Collection 6 burned-area data to scale burned-area estimates (*28*), field observations to constrain fuel consumption (*29*), and emissions factors compiled in the Global Fire Emissions Database (GFED4s) (*13*). Our results indicate that deforestation fires were the dominant Amazon fire type from April to December 2019 in terms of total VIIRS fire detections and estimated carbon emissions. Time series of fire activity by fire type underscore the potential to use fire type information to mitigate ecosystem and economic impacts in future fire emergencies.

## RESULTS

The VIIRS sensors detected nearly 1.4 million active fire pixels within the Amazon biome during the Southern Hemisphere fire season in 2019 (Table 1). A total of 19,700 deforestation fires accounted for 39% of VIIRS fire detections, as each deforestation event triggered an average of 28 fire detections. Approximately 3000 understory forest fires were responsible for an additional 12% of fire detections. Small clearing or agricultural fires were the most numerous (113,000) but accounted for only 17% of all fire detections, while savanna fires accounted for the remaining 32%.

**Table 1.** Active fire detections, fire size, and carbon emissions by fire type for the southern Amazon and countries in the Southern Hemisphere study region during the 2019 fire season. We classified fires with ≥50% tree cover as deforestation, forest, or small clearing and agricultural (“small”) fires, while fires in more open cover types (<50% tree cover) were classified as savanna and grassland fires (fig. S3). Mean event size, total burned area, and fire carbon emissions for deforestation, small, and savanna fire types are based on scaled estimates of fire size (see Materials and Methods). Large, fast-moving savanna fires may be fragmented by the clustering algorithm (Fig. 1D), leading to an overestimate in the number of fire events and a corresponding underestimate of mean fire size, despite robust estimates of total burned area and fire emissions.

**Region**	**Fire type**	**Fire detections (×1000)**	**Mean fire radiative power (MW)**	**Number of events (×1000)**	**Mean event size (km^2^)**	**Burned area (×1000 km^2^)**	**Emissions (Tg C)**
Amazon	Deforestation	547.06	16.10	19.71	0.64	12.52	68.90
	Forest	163.10	10.61	3.04	4.98	15.13	19.01
	Small	247.83	11.14	113.03	0.05	5.59	7.64
	Savanna	456.21	11.04	76.77	0.52	39.82	14.50
Brazil	Deforestation	609.38	15.52	20.32	0.77	15.62	80.76
	Forest	244.26	12.05	3.37	7.52	25.34	32.71
	Small	215.12	11.45	95.76	0.05	4.86	6.65
	Savanna	1451.27	11.97	224.53	0.68	153.12	55.26
Paraguay	Deforestation	22.77	15.23	0.55	2.51	1.38	5.05
	Forest	19.13	19.86	0.25	9.14	2.26	3.98
	Small	14.46	9.42	4.70	0.06	0.27	0.37
	Savanna	130.27	11.72	16.73	0.88	14.78	5.33
Bolivia	Deforestation	100.53	12.78	2.14	1.22	2.6	11.93
	Forest	370.71	12.84	1.48	21.71	32.07	48.41
	Small	48.59	10.41	20.08	0.05	1.04	1.40
	Savanna	228.19	13.88	24.57	1.06	26.15	9.62
Peru	Deforestation	0.49	7.41	0.02	0.07	0.00	0.01
	Forest	0.41	7.97	0.02	2.04	0.04	0.04
	Small	3.65	7.54	2.14	0.04	0.09	0.11
	Savanna	15.96	8.03	6.57	0.06	0.41	0.15
Southern hemisphere South America (0°–25°S)	Deforestation	756.42	15.15	24.24	0.82	19.77	99.18
	Forest	637.58	12.73	5.28	11.39	60.09	85.46
	Small	348.48	10.91	154.67	0.05	7.77	10.55
	≥50% tree cover	1742.49	13.42	184.19	0.48	87.63	195.19
	<50% tree cover	1909.51	12.22	296.36	0.67	198.00	71.74

Clustering VIIRS detections into individual deforestation, forest, and small fire events provided valuable context beyond simply tracking fire detections. Each fire event includes an estimated ignition location, start date, fire duration, and carbon emissions. Fire perimeters for deforestation and forest fires also provide an approximate estimate of the area burned (Fig. 1, A and B), and the preliminary estimate of the fire-affected area from the clustered fire detections can be calibrated to quantify fire emissions for each event. We scaled burned area from savanna and deforestation fires to match 500-m burned area from MODIS (*27*) (see Materials and Methods) and used a single scaling factor (0.1) to estimate the burned area from isolated clearing and agricultural fires that are typically much smaller than the 375-m resolution of the VIIRS imagery (Fig. 1D and Table 1) (*26*). Because of challenges related to mapping burned area under dense canopy with algorithms designed to detect the spectral signature of char or ash (figs. S1 and S12), we used the unadjusted fire perimeters derived from active fire clusters in this study to estimate area burned from forest fires. Forest fires were the largest fire type, with a mean size of 5.0 km^2^ that was about eight times the size of the average deforestation fire event (0.6 km^2^). Numerous small clearing and agricultural fires accounted for only 8% of total burned area in the Amazon biome, while savanna and grassland fires accounted for nearly 55% of total burned area. This information on individual fire type and attributes provides important context on the spatiotemporal evolution of Amazon fire activity and fire carbon emissions.

**Fig. 2.**
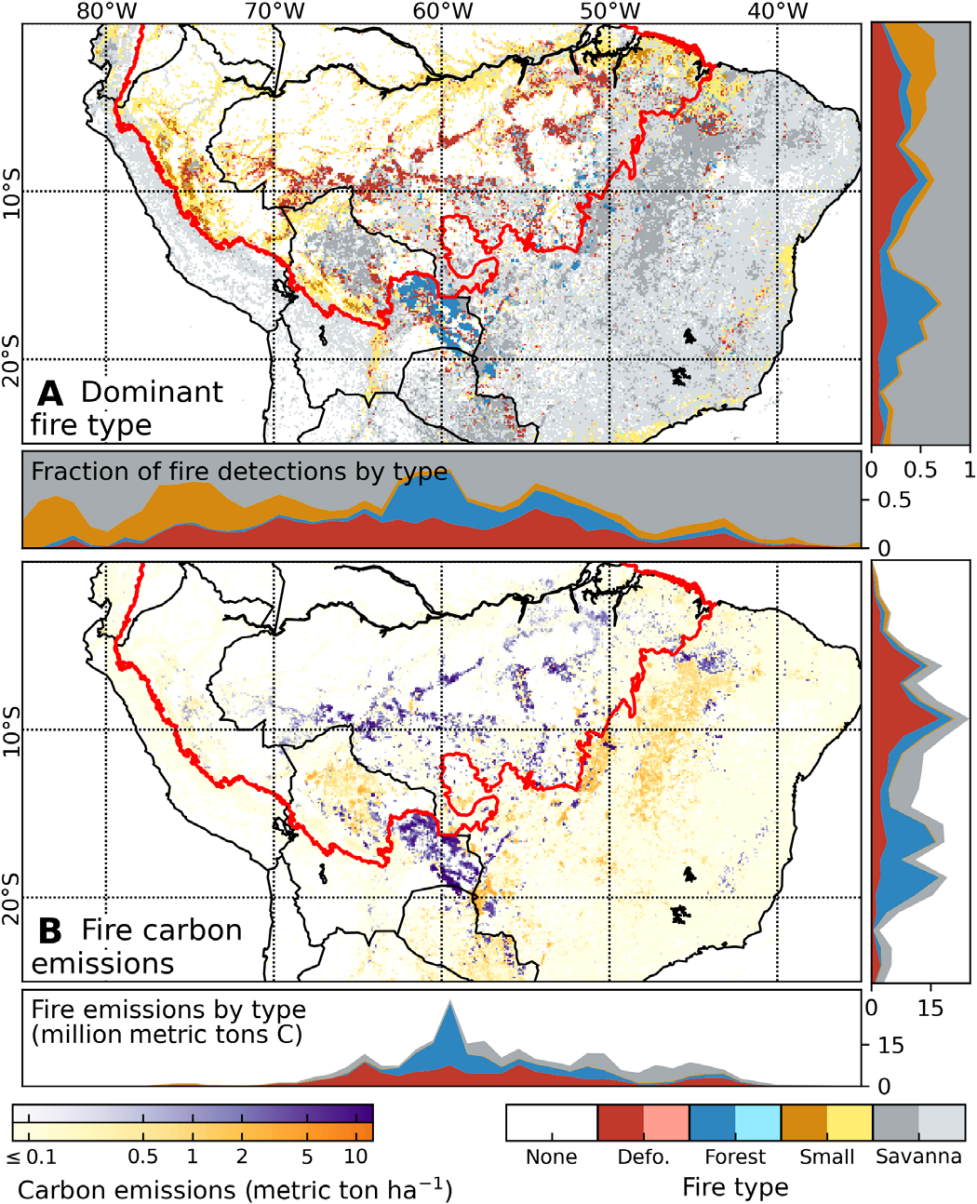
Separation of deforestation and forest fires from other fire types identifies regions with long-term carbon losses from fire. (**A**) Dominant fire type and (**B**) estimated carbon emissions during April to December 2019 at 0.1° resolution. In (A), grid cells with fewer than 50 fire detections are shown in lighter shades of the same color. In (B), long-term carbon losses from deforestation and forest fires are shown in purple; short-term carbon losses from small clearing and agricultural fires and savanna and grassland fires are shown in orange. Insets show the contribution of different fire types per 1° bin of latitude and longitude (see fig. S15 for more details about the Amazon biome).

Our analysis also revealed complex regional fire patterns that contributed to air quality impacts from fire emissions across a larger domain in South America between the equator and 25°S (Fig. 2). Deforestation fires were the dominant fire type across the arc of deforestation in Brazil and hot spots of agricultural expansion in the Brazilian Cerrado, along with some forested regions of Peru, Bolivia, and Paraguay. Even outside of the Amazon biome, fires in areas with >50% tree cover can be classified as deforestation fires on the basis of the pattern of repeated, high-intensity burning (figs. S3 and S4). Forest fires were the dominant fire type in Bolivia, where an estimated 1480 understory fires burned 32,000 km^2^ of Amazon and Chiquitania forest during the 2019 dry season. Forest fires also accounted for an important fraction of all fire detections in Brazilian states with dense Cerrado vegetation or drier Amazon forests. Small clearing and agricultural fires were the dominant fire type across regions with infrequent burning, including wetter Amazon forests in Peru and along the main stem of the Amazon River in Brazil. Last, about one-half of all active fire detections across the larger South American study region were classified as savanna and grassland fires (Fig. 2).

Separation of fire carbon emissions by fire type provides insight into the contributions from 2019 fires in the Amazon region to the global carbon cycle (Fig. 2B). In particular, carbon emissions from deforestation (69 Tg) and forest (19 Tg) fires contribute to long-term changes in atmospheric concentrations of greenhouse gases. Although emissions from forest fires were comparatively small, our estimates of forest fire emissions exclude larger committed carbon losses from tree mortality ranging from 30 to more than 50% of total aboveground biomass (*14*, *30*). Savanna (15 Tg) and small clearing and agricultural (8 Tg) fires also contributed to total carbon emissions and regional impacts on air quality, but these fire events account for much smaller net carbon emissions than forest fire types based on rapid regrowth of herbaceous vegetation in the following wet season.

**Fig. 3.**
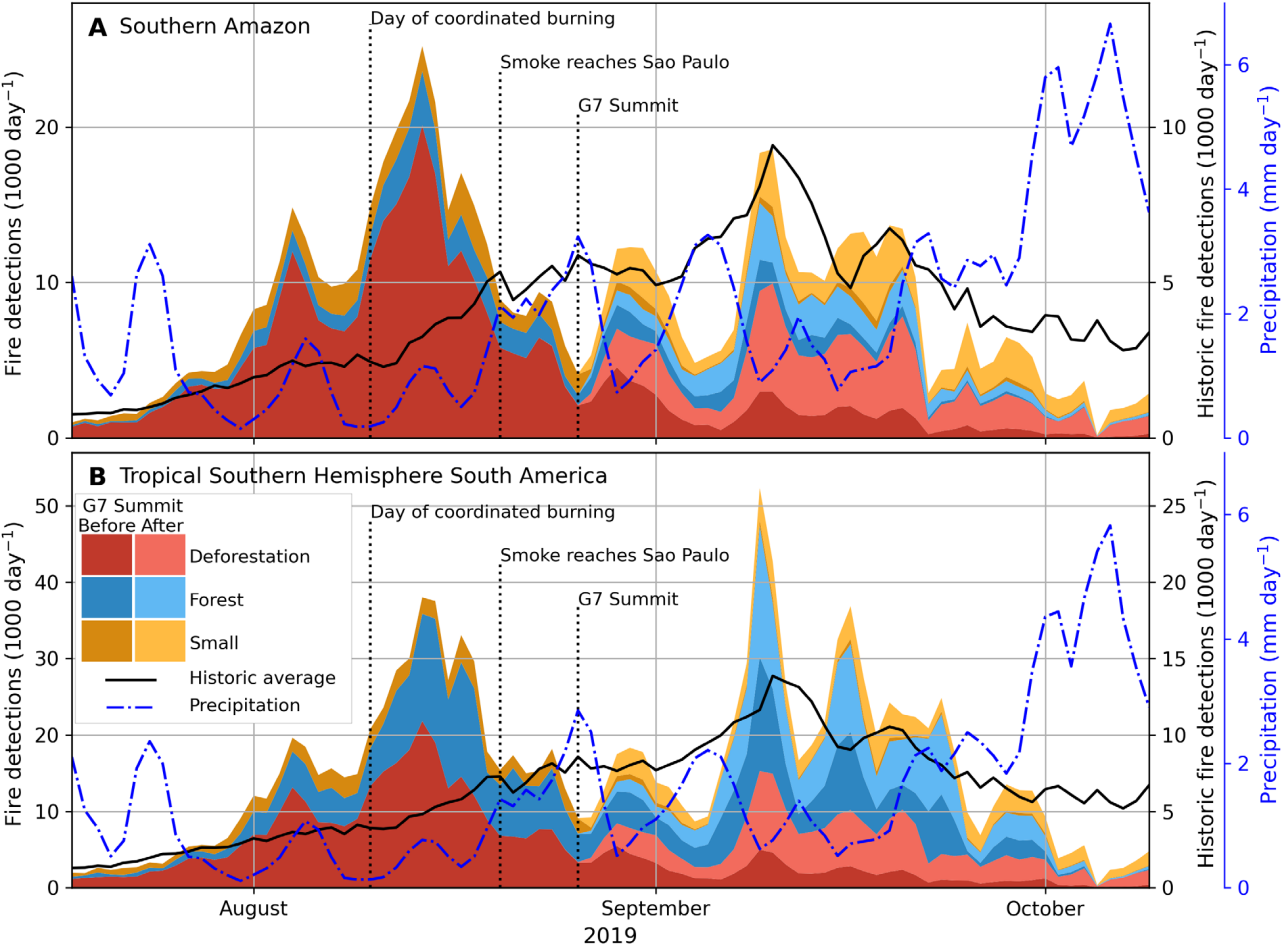
Deforestation caused anomalous fire activity early in the 2019 Amazon fire season. Daily fire detections in forest ecosystems (tree cover greater than 50%) classified into three fire types for (**A**) the southern Amazon and (**B**) tropical southern hemisphere South America (0° to 25°S; see Fig. 2). Light shades highlight the contribution from new fires started after the G7 Summit on 24 to 26 August. The solid black line (right *y* axis) indicates historic average (2012–2018) daily fire detections based on a single VIIRS sensor, and the intermittent blue line (second right *y* axis) shows mean daily precipitation across all 0.1° grid cells with 50 or more VIIRS fire detections in 2019.

Deforestation fire activity in the southern Amazon increased rapidly at the end of July 2019 (Fig. 3), with a peak in deforestation fire detections (>20,000 day^−1^) shortly after the coordinated day of burning in the Brazilian state of Pará (*31*). Satellite detections of deforestation and other fire types were already declining by the time the Amazon smoke plume reached São Paulo, Brazil, and declined further in the lead up to the G7 summit of world leaders. Total fire activity after the G7 summit remained at or below the average fire activity from 2012 to 2018, especially in Pará (fig. S15), consistent with regional fire suppression efforts and policy interventions in response to global attention to Amazon fires in 2019 (*32*). Nevertheless, new deforestation fires started after August 26 added 23 Tg C emissions, or 34% of the total deforestation fire emissions from the southern Amazon in 2019.

Rainfall was the dominant driver of temporal variability in 2019 Amazon fire activity. The effect of rainfall on fire activity is clear and immediate, as rainfall patterns synchronize regional variations in flammability. In all major southern Amazon biomass burning regions, the increase in rainfall in late August and early September led to sharp declines in fire activity (fig. S16). Forest fires accounted for more than half of all active fire detections for the larger study region in September and contributed disproportionally to the peak day of forest fire detections (>50,000 day^−1^) on September 12. About half of the area affected by forest fires occurred in Bolivia (Table 1), where fires burned for months in remote forests (fig. S17), and the total area affected by forest fires (85,500 km^2^) nearly equaled the area affected by deforestation fires (99,200 km^2^) across the Southern Hemisphere study region, in contrast to previous reports of burning predominantly in existing cleared areas (*23*). Last, widespread rainfall across the region during mid-September and early October extinguished understory fires and brought the fire season to an early end in 2019 compared to previous years (2012–2018).

Amazon fire types can be accurately identified in near real time using patterns of fire detections and land cover information. On the basis of estimates of 2019 deforestation in the Brazilian Amazon (*6*), we were able to accurately identify 67% of active fire detections (fig. S6) associated with 2019 deforestation in Brazil after the second day of a new fire start. These accuracies improved to 80% for fire detections after 7 days. Our dataset also identified active fire detections from deforestation fires not mapped as deforestation (26%; fig. S8). These fires represent burning likely associated with deforestation in secondary forests or new clearing activity after the cutoff dates for annual deforestation assessments from Project for Monitoring Amazon Deforestation (PRODES). Our classification algorithm also showed that most of the forest fires were not associated with 2019 deforestation, suggesting that these fires originated from other ignition sources such as small but numerous management fires (fig. S9). A comparison against independent fire type data from MAAP (2099 large fire events) revealed strong agreement, with our approach correctly identifying 75% of deforestation, 69% of grassland, and 64% of forest fire events from the MAAP dataset (fig. S10). A lower percentage of active fire detections classified as deforestation (57%), savanna (18%), and forest (47%) in our study had the same classification in MAAP, for example, because the MAAP dataset includes a separate “cropland and pasture” fire class that overlaps with the savanna fire type in our classification. Differences between datasets therefore reflect both errors of omission and commission along with differences in the definitions of the various fire types. A further quality assessment based on 10-m-resolution pre- and postfire Sentinel-2 image pairs revealed that our algorithm was able to separate deforestation from forest fires events with 66% accuracy, increasing to 92% accuracy for fire detections (table S2; see the Supplementary Materials for further details).

**Fig. 4.**
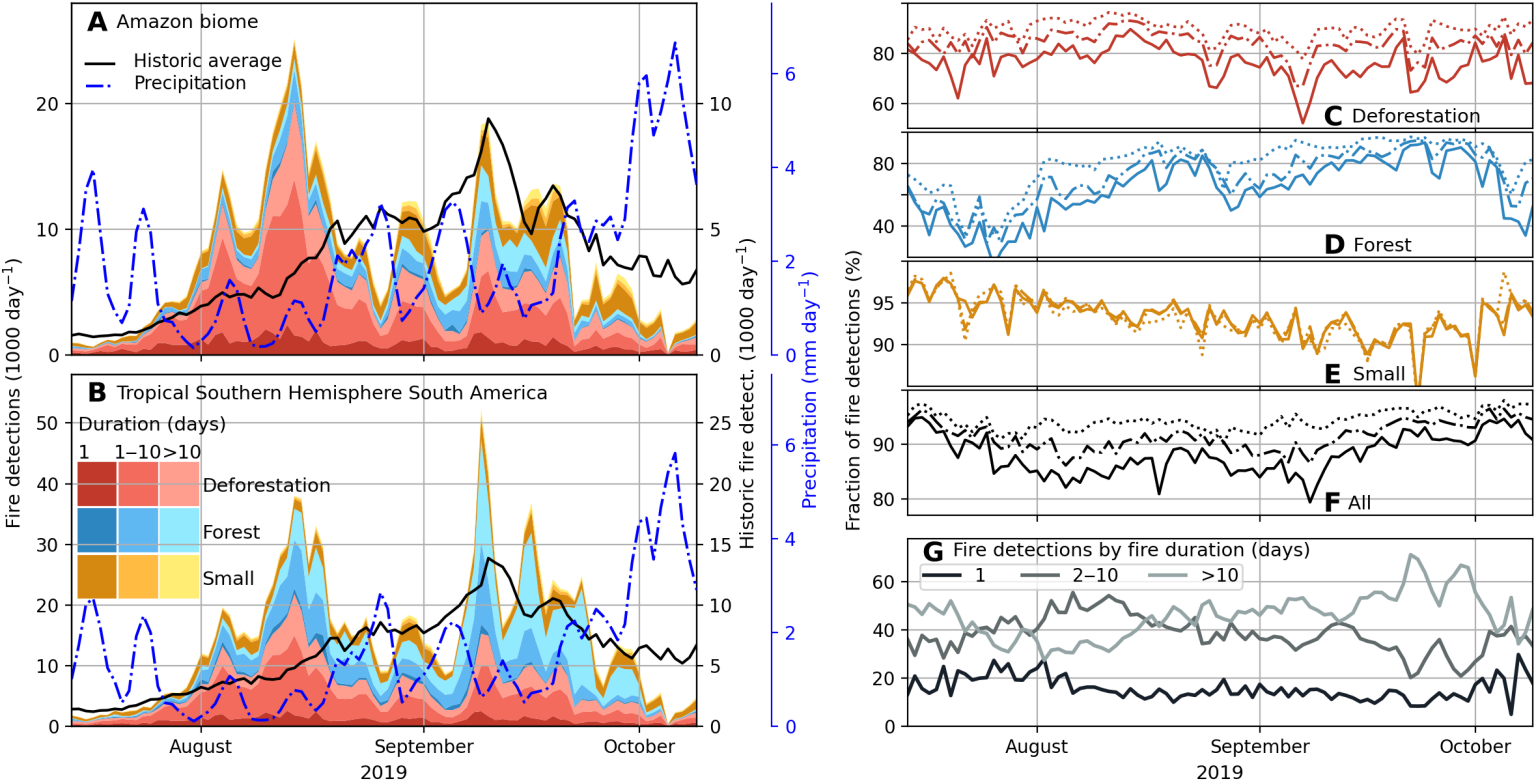
Accurate classification of satellite active fire detections by fire types is possible in near real time based on unique characteristics of land cover and fire behavior, including the long duration of deforestation and forest fire types. Daily fire detections from new fire starts (dark shades) and fires that burned between 1 and 10 days or longer than 10 days (lighter shades) in forest ecosystems classified into three fire types for (**A**) the southern Amazon and (**B**) tropical southern hemisphere South America (0° to 25°S). (**C** to **F**) Daily fraction of active fire detections attributed to their final end of season fire type, for 1- (solid), 2- (dot dash), and 7-day (dotted) lag times, for (C) deforestation fires, (D) forest fires, (E) small clearing and agricultural fires, and (F) all fire types combined. (**G**) Daily fraction of satellite active fire detections from fires burning for 1 day, 2 to 10 days, and >10 days, respectively.

The long duration of most Amazon fires aids near–real-time classification, as only 14% of daily fire detections were associated with new fire starts during the middle of the fire season (Fig. 4). During August and September, 76% of all detections associated with deforestation fires were correctly classified on the day of detection and more than 95% of all fire detections were correctly classified by fire type within 1 week. The importance of multiday fires for the total burned area (81%) and fire emissions (92%) from the southern Amazon region in 2019 also underscores the value of early detection and fire type information. Near–real-time assessments of individual fire locations by fire type could revolutionize regional fire management strategies and improve scientific understanding of fire-climate interactions to support Amazon forest conservation efforts.

## DISCUSSION

Fire is an important landscape management tool across the Amazon biome (*12*), but expansion of the agricultural frontier (*12*) and an increasing frequency of drought conditions (*33*) have elevated the risk of fire-driven degradation of Amazon forests. Improving fire management is therefore essential to counter this threat, promote sustainable development in the region, and achieve global climate change mitigation objectives, including the Paris Agreement. Here, we developed a new approach that uses VIIRS active fire detections to cluster and classify fire types in near real time and estimate fire carbon emissions associated with individual fire events. An initial quality assessment revealed strong agreement with independent deforestation data, fire types from the MAAP project, and reference data from visual interpretation of Sentinel-2 image pairs. Classification accuracy increased for larger fires with higher carbon emissions, while small fires, or fires burning through multiple cover types, were more challenging to classify accurately.

Deforestation fires were responsible for most carbon emissions early during the 2019 fire season (*23*), but extensive forest fires in the southern Amazon and Bolivia contributed most emissions during September and October. Our results represent a step forward in terms of near–real-time emission attribution, as existing inventories (*13*, *34*) do not account for different fire types within the Amazon region, such as the separation of deforestation and forest degradation fires. The classification into new fire starts and different fire types confirms the importance of deforestation fires to overall fire activity and emissions in 2019 and provides the basis for more targeted responses to future fire emergencies in the region.

## MATERIALS AND METHODS

NASA’s MODIS instruments on board the Terra and Aqua satellites have provided routine daily estimates of global fire activity since 2000 (*22*). Nevertheless, the relatively coarse resolution of the MODIS thermal imagery, with effective pixel resolution increasing from 1 km^2^ at nadir to about 10 km^2^ at off-nadir angles, leads to an underestimate of total fire activity, especially for low-energy fires (*35*, *36*). Together, the VIIRS instruments on board Suomi NPP (launched in 2012) and NOAA-20 (launched in 2017) improve global coverage for fire detection with higher spatial resolution of individual fire detections (0.14 km^2^), more accurate geolocation information, and more consistent fire detection sensitivity across the full image swath.

In this study, we combined active fire detections from both VIIRS instruments to map the extent of individual fires using the Global Fire Atlas algorithm (*25*). For each fire event, we combined attributes of adjacent and sequential daily fire detections to estimate fire behavior. These metrics of fire behavior were then combined with data on vegetation carbon stocks and historic land use for rapid identification of specific fire types. We used an expert-guided approach for fire type classification because no consistent training data were available, and the separation of fire events into four distinct classes matches regional information needs for fire management and emission estimation. We assessed the quality of our fire type classification using independent data on 2019 deforestation and fire type information for a subset of Amazon fires from MAAP and Sentinel-2 image pairs to evaluate the performance of both the end-of-year product and the near–real-time approach. To better understand the relative contribution of different fire types to regional greenhouse gas emissions, we scaled VIIRS active fires to fire carbon emissions using the MCD64A1 collection 6 burned-area dataset (*28*), fuel consumption estimates from field observations (*29*), and emissions factors from GFED4 (*13*). Last, we provide a regional analysis of the evolving 2019 fire season and its drivers.

### Tracking individual fires based on VIIRS data and the Global Fire Atlas approach

The Global Fire Atlas uses a new algorithm to track the daily development of individual wildfires and derive a number of metrics on fire behavior, including fire size, duration, and rate of spread (*25*). The Global Fire Atlas algorithm was initially applied to the MCD64A1 collection 6 burned-area product, a daily estimate of global burned area at 500-m resolution (*28*). However, burned-area data have two important limitations compared to active fire detections for the development of a near–real-time algorithm to track fires across the Amazon basin. First, burned-area data are not available in near real time, as the MCD64A1 algorithm relies on time series of data before and after the fire, leading to a 2- to 3-month delay in the production of the global product. Second, coarse-resolution (500 m) satellite observations are often unable to accurately map the burned area associated with small fire types or low-intensity forest fires burning beneath a dense forest canopy (*24*). By contrast, active fire detections are available in near real time, and previous studies have demonstrated that active fire data can provide a robust estimate of burned area in forested ecosystems based a persistent thermal signal within each larger grid cell from the slow spread rates and residual smoldering (*37*, *38*).

Here, we combined 375-m active fire detections (*26*) from the Suomi-NPP and NOAA-20 VIIRS instruments to map individual forest fires across tropical southern hemisphere South America (between 0°S and 25°S). We analyzed VIIRS data available through NOAA for April to December 2019, including fire detections in all confidence classes. This interval started well before the onset of the southern hemisphere burning season and extended past the onset of the wet-season conditions. Even though NOAA-20 was launched in 2017, fire detection data only became available starting in April 2019.

Combined, the two VIIRS instruments provide three major improvements compared to the MODIS instruments on board NASA’s Terra and Aqua satellites. First, the VIIRS 375-m product is more sensitive to small and low-intensity fire types because of the higher spatial resolution and enhanced nighttime sensitivity (*26*). This improved sensitivity increases the probability for early detection of new fire starts and offers a more complete picture of total fire activity, including agricultural and forest fires. Second, the VIIRS instruments maintain a more consistent pixel area across a wider swath (*39*), improving the ability to detect and locate low-energy fires, even at off-nadir view angles, and reducing coverage gaps across the tropics (*40*). Better spatial resolution and geolocation information of VIIRS 375-m active fire detections also improves the accuracy of land cover information associated with each fire location. Third, the constellation of both VIIRS instruments provides global daily daytime and nighttime observations at near-nadir observational angles; near-nadir observations have a shorter atmospheric path length and less interception by the canopy layer, minimizing signal loss to retain sensitivity to smaller fire events and understory burning in Amazon forests (*41*).

To map individual fire perimeters from VIIRS active fire detections, we first gridded active fire detections at a 0.005° (~550 m) resolution based on the center location of each fire pixel (fig. S1). We selected this spatial resolution to accommodate typical understory forest fire spread rates of 100 to 400 m day^−1^ (*30*, *42*) and reduce potential effects of any residual geolocation error on derived estimates of fire persistence, an important indicator of deforestation activity (*12*, *43*). To prepare the data for the Fire Atlas algorithm, we selected the earliest active fire detection within each 550-m grid cell. Following the Global Fire Atlas approach, we then applied a spatial filter to remove inconsistencies in the estimated burn date within each fire to identify the ignition location (*25*). Because fire use is widespread across the tropics, an additional threshold is required to separate adjacent fires that burned at different times during the fire season. This threshold sets the maximum number of days for a single contiguous fire to spread into an adjacent grid cell; here, we set this threshold as ≤5 days after the last active fire detection within any given 550-m grid cell. For example, in fig. S1 (C and D), a deforestation fire in an adjacent field burned into the understory of neighboring forest area, resulting in fire detections adjacent to our example fire on day 220, 8 days before the example fire was ignited. In this case, the algorithm successfully classified these as two separate fire events despite their spatial proximity. In line with previous work (*25*, *38*), we found that savanna fires typically spread several kilometers per day, resulting in artificial fragmentation of individual events on our finer 550-m grid (e.g., see Fig. 1C). We therefore used land cover data to distinguish savanna from forest fires across the region. The Fire Atlas algorithm is computationally efficient and can be applied on a daily basis to track individual fire perimeters in near real time as new active fire data becomes available.

Thermal anomalies detected from space are most often fires, but these products also capture other features that are hotter than their surroundings such as volcanoes, gas flares, and industrial activity (*22*, *26*). To remove the influence of static sources on our analysis, we excluded 224 fire events (47,128 fire detections) containing 550-m grid cells with more than 20 active fire detections in at least three of seven historic fire years (2012–2018) based on the VIIRS sensor on board Suomi-NPP. On the other hand, clouds or dense smoke may reduce the ability of the VIIRS instruments to detect active fires. Although this will affect the absolute numbers of active fire detections on any given day, the persistent (multiday) fire signal within each larger grid cell in this analysis mitigates the influence of unobserved fire activity on the estimated extent of each fire. The combination of both VIIRS instruments also reduces the effect of cloud cover or smoke on daily fire detections because the instruments are spaced 50 min apart and observe the same fire at different view angles. Therefore, the VIIRS data in this study are not corrected for cloud cover, because statistical models used in other studies to account for cloud cover cannot be easily attributed to specific fire events [e.g., (*34*)].

### Fire type classification: Selection of training data

We combined attributes of individual fire events derived from the Global Fire Atlas algorithm with existing land cover and land use change information to classify each fire as one of four fire types: (i) deforestation fires, (ii) forest fires, (iii) small clearing and agricultural fires, and (iv) savanna and grassland fires (Fig. 1). Savanna and grassland fires in both natural vegetation and managed lands were separated on the basis of a simple tree cover threshold (<50%) within the fire-affected area. The remaining three fire types all occur in landscapes with ≥50% tree cover and therefore cannot be separated on the basis of land cover data alone. We used a training dataset of deforestation and forest fires to develop a multivariate classification approach to separate deforestation, forest, and small clearing and agricultural fires.

We used deforestation data from the PRODES from the Brazilian National Institute for Space Research (*6*) to explore the relationship between historic deforestation in the Brazilian Amazon and associated fire activity. PRODES deforestation estimates are produced annually using 30-m resolution Landsat and other high-resolution imagery and available within 1 year of the end date for image collection. Here, we compared the VIIRS active fire detections and mapped deforestation polygons on the regional 550-m grid (fig. S2). Within each 550-m grid cell, we attributed all active fire detections associated with deforestation activity to the year with largest extent of deforestation registered by PRODES. Consistent with previous studies (*12*, *43*), we found elevated fire activity up to 5 years after the year in which deforestation was initially mapped, based on the repeated use of fire to remove woody debris after initial clearing (fig. S2). On the basis of this finding, all individual fire events identified by our algorithm with ≥25% of 550-m grid cells associated with historic deforestation (2014–2018) were classified as deforestation fires. In addition to evidence for multiple years of fire activity after the year of deforestation, historic deforestation data also provide a strong indicator of active deforestation frontiers and hence the likelihood of new deforestation and associated fires in 2019. We used this threshold of ≥25% overlap with historic deforestation (2014–2018) to create a dataset of 12,039 deforestation fire events in 2019 to identify their characteristics.

Deforestation and forest fires are particularly challenging to separate, because both fire types may exhibit similar patterns of persistent burning across large forested areas. We therefore manually selected 77 forest fires across both the Amazon biome and the larger study region, tropical southern hemisphere South America (0° to 25°S), to identify unique characteristics of this fire type (table S1). We selected only large forest fires to train the classification for two primary reasons. First, contiguous areas of recent deforestation typically do not exceed 50 km^2^, while large forest fires can easily exceed 100 km^2^. Second, large forest fires typically develop circular patterns of fire progression based on well-developed fire fronts (fig. S1A) (*14*) that can be easily identified through visual interpretation in standard Geographic Information System (GIS) software. For all selected forest fires in the Brazilian Amazon, less than 5% of 550-m grid cells within each larger understory forest fire event contained historic deforestation (2014–2018) based on PRODES data.

### Fire type classification: Identifying thresholds of fire type characteristics

We used three types of information about each fire event to rapidly identify fire types and assign confidence intervals (fig. S3). First, we used data on land cover and historic land use, including fractional tree cover, historic deforestation, and a pantropical biomass map. To accommodate delayed effects of tree cover losses on fire, we used estimated forest cover in 2014 to separate savanna from forest fire types, calculated on the basis of the difference between the 2000 fractional tree cover map and 2000–2013 tree cover losses from the Global Forest Change dataset at 30-m resolution (*10*). Historic deforestation (2014–2018) was estimated on the basis of 30-m-resolution PRODES data available for Brazil (*6*). We used a pan-tropical biomass map at 1-km resolution (*44*), a product developed from the fusion of two existing biomass maps (*45*, *46*) with additional biomass training data. We combined these data with grid cell–level fire characteristics, such as fire persistence and fire radiative power (FRP), and multiday metrics of fire events such as fire size and total fire detections (figs. S3 and S4). Gridded metrics, such as fire persistence (calculated for each 550-m grid cell), were averaged across all grid cells within each fire event, while metrics per fire event, such as FRP, were based on equal weight of all satellite fire detections within the fire perimeter. Together, these metrics provide a robust path to classifying fire events by fire type. To mimic near–real-time classification of the active fire data, all variables were computed on a daily basis during the 2019 study period to track the evolution of individual fire events.

Deforestation fires were identified on the basis of historic maps of deforestation and differences in fire behavior compared to other fire types (fig. S3). Fires containing ≥25% of grid cells with overlapping historic PRODES deforestation during 2014 to 2018 were classified as deforestation fires with high confidence and used to characterize typical deforestation fire behavior (fig. S4). For all other large fires (>5 fire detections and persistence >1) with ≥50% forest cover, we developed a multivariate approach to separate fire activity from deforestation and forest fire events. On the basis of the subset of reference fires, we selected five indicators of fire behavior to separate deforestation from forest fires (figs. S4 and S5). In addition, we used a threshold of 120 metric ton ha^−1^ biomass to select between metrics of fire behavior for forest fires in moist versus dry forest types. In high-biomass Amazon forests, deforestation fires consistently have higher FRP than forest fires, allowing for detection of deforestation fires with high confidence. In lower-biomass forests, typical of drier regions, forest fire behavior was more similar to savanna fires, with higher average FRP, lower fire persistence (based on faster spread), and a pronounced diurnal cycle resulting in a larger fraction of daytime fire detections. In low-biomass forest systems, deforestation fires were therefore not easily separable from forest fires using FRP. Instead, high-confidence deforestation fires were distinguished on the basis of higher fire persistence. Deforestation fires were also typically small compared to forest fires, and we therefore included fire size as an additional indicator of fire type. Fires smaller than 40 km^2^ were more likely to be associated with deforestation, whereas fires larger than 100 km^2^ were classified as high-confidence forest fires. For those fires that could not be directly classified as either deforestation or forest fire on the basis of these primary indicators, we combined all five metrics to estimate the fire type with three different confidence levels. For each indicator, we set a threshold suggesting either deforestation or forest fire activity; if all five metrics indicated deforestation, we assigned five points resulting in a high-confidence deforestation fire. At the other extreme, if all five metrics indicated a forest fire, then the total of points would be zero, resulting in high-confidence forest fire (see purple box in fig. S3).

Small fires for clearing and agricultural management were identified on the basis of the small total number of active fire detections (≤5) and low fire persistence (1 day), consistent with fast-spreading fires in herbaceous or other low-biomass fuel loads or short duration. Improved geolocation of the VIIRS 375-m active fire data enables a more robust combination of fire location with land cover data to rapidly identify fires burning in open-cover types. Last, we used fractional tree cover data to assign all fires with a majority of fire-affected area in landscapes with <50% tree cover to savanna fires with high (<20% tree cover), medium (20 to 40% tree cover), and low (40 to 50% tree cover) confidence. All fire type estimates were based on daily per-fire averages, such that a fire that started in a savanna adjacent to forest cover would be classified as a forest fire once the average tree cover across all grid cells within the perimeter exceeded 50%.

### Fire type classification: Quality evaluation

We evaluated the 2019 fire type classification using four different approaches. First, we compared our classification to independent maps of 2019 deforestation. We assessed the time needed to accurately identify new deforestation fires (fig. S6) and explored the sensitivity of the end-of-year classification of deforestation fires to the availability of historic deforestation data (fig. S7), data that were only available for the Brazilian Amazon. For this evaluation, we classified all 4984 fires with ≥25% of grid cells overlapping with 2019 deforestation with and without the use of historic deforestation data. In addition, we compare the overlap between deforestation fires, forest fires, and observed deforestation rates from PRODES for 2014 to 2018 and 2019 separately, to explore data quality and understand the underlying causes of forest fires (figs. S8 and S9). Second, we generated data for the 2020 fire season using the same classification approach (fig. S3) to compare our fire type classification to independent data from the MAAP project that was only available for 2020 (*27*). We selected all fires (2099) from the MAAP database for Brazil that had overlap with fires identified in our study. MAAP classified fires that produce substantial smoke and aerosols into four distinct classes, “deforestation fires” following forest clearing, “forest fires,” “cropland and pasture fires,” and “grassland fires” on the basis of expert-guided interpretation of various satellite data products including high-resolution commercial imagery. We weighted these comparisons by the number of active fire detections associated with each fire. The results provide a first estimate of omission and commission errors, with close agreement between datasets for more similar fire types and less agreement for fire types with different definitions (fig. S10). Third, we assessed the classification accuracy for the separation of deforestation and understory forest fires, the two primary types of multiday fires in the Amazon, and a critical distinction for accurate estimates of carbon emissions from deforestation and forest degradation (table S2). Because of the class imbalance, we selected a stratified random sample of 100 deforestation and 100 understory fires across the South American study domain in 2019. We focused on fires that started in August, to avoid issues of cloud cover, and used pre- and postfire Sentinel-2 images at 10-m resolution to interpret the reference fire type. Fourth, we investigated how well we were able to identify different fire types in near real time, as compared to our final, end-of-year classification (Fig. 4).

### Estimating fire carbon emissions

In this study, we combined our fire classification with estimates of burned area (square meters) and fuel consumption (grams per square meter) to derive estimates of total dry matter burned (grams) and carbon emissions per fire. First, we translated fire perimeters derived here to estimates of area burned (fig. S11). On average, the MCD64A1 collection 6 burned-area product performs well for large continuous burned areas (*28*, *47*), including savanna ecosystems or fires following large deforestation events. In contrast, moderate-resolution satellite data may not capture small fires (*47*), or fires burning under dense canopy (*24*). Therefore, we scaled burned area for savanna and deforestation fires to the MCD64A1 burned-area data but used separate scaling factors for small clearing and agricultural fires and forest fires. First, to scale burned area from savanna and deforestation fires separately, we identified the fire type of each cluster of continuous burned area (adjacent 500-m burned pixels) within the MCD64A1 burned-area product for 2019. For each continuous cluster of MCD64A1 burned area, we determined the fire type on the basis of the overlap with fire events based on clustering of active fire detections in this study and used the dominant fire type within each cluster (largest overlapping area). A small fraction of total MCD64A1 burned area (about 0.7%) did not have overlap with fires detected in our study, usually short-lived fires in low-fuel systems. Second, we derived burned-area scaling factors for savanna and deforestation fires per grid cell based on the ratio between MCD64A1 burned area and the total pixel area (at ~550-m resolution) within the perimeters of individual fire events identified by our approach. An iterative region-growing analysis initially estimated scaling factors for all 0.5° grid cells that contained at least 50-km^2^ burned area from both MCD64A1 and the area within the fire perimeters derived here. Next, we clustered remaining 0.5° grid cells that did not meet the criteria to 1° and evaluated the larger regions using the same fitting criteria. The grid cell aggregation and evaluation were repeated at 1°, 2°, and 4° resolutions before all remaining grid cells were combined into a single continental-scale region (fig. S11). We found overall good performance of MCD64A1 burned area for forest fires in the Xingu region (e.g., fig. S1) and other forested areas that might have burned previously. However, considerable omission occurred for fires in areas of dense forest that had not burned since 2000 (fig. S12). Because the combination of active fire detections from the VIIRS instruments on board Suomi-NPP and NOAA20 typically resulted in dense point clouds of active fire detections for forest fires (fig. S12; on average >10 active fire detections per square kilometer of burned area), and the average forest fire size was large (11.4 km^2^), we used our estimate of burned area associated with forest fires without further adjustments. For small clearing and agricultural fires, we used a scaling factor of 0.1; a comparison to Landsat-derived burned-area data for 2019 from MapBiomas for small clearing and agricultural fires indicated that this was a reasonable scaling factor.

We evaluate burned-area estimates by comparing results to Landsat burned-area estimates from the MapBiomas (*48*) for Brazil (fig. S13). First, we classified the MapBiomas burned area into burned area from different fire types using the same approach as used for the MODIS data. Continuous clusters of burned area were overlayed with fire perimeters derived here, and their fire type was assigned on the basis of the fire type that had most overlap with the burned area clusters. Burned-area clusters that remained entirely undetected by our approach were assigned to the savanna class if average tree cover was below 50% (consistent with our approach) or to a “residual” class if tree cover was above 50%.

In addition to burned area, carbon emissions from fires depend on fuel consumption (grams per square meter), the product of aboveground biomass and combustion completeness. Building on the development of existing near–real-time fire emission inventories (*34*, *49*, *50*), we developed conversion factors between VIIRS active fire detections and fuel consumption to estimate 2019 carbon emissions for each fire in the study region. These conversion factors scale FRP to dry matter burned, a required step because satellite observations provide estimates of instantaneous energy release (FRP), but how this translates into total fire radiated energy and emissions is further modified by instrument characteristics (*51*), the fire diurnal cycle (*52*), vegetation (*41*), and cloud cover (*34*). We derived conversion factors by fitting observed distributions of fuel consumption (metric tons per hectare) during field experiments (*29*) to the observed total of FRP per area burned (megawatts per hectare; fig. S14). This approach resulted in average fuel consumption estimates that are close to field observations of each fire type but distributes fuel consumption per fire based on the cumulative FRP. For savanna fires, we included fuel consumption from eight field observations including two observations for Campo limpo, Campo Sujo, Cerrado aberto, and Cerrado denso, the dominant savanna types (*53*, *54*). For deforestation fires, we included 19 field measurements associated with fires following tropical forest clearing in Brazil [summarized in (*29*)]. In our classification approach, small clearing and agricultural fires are associated with ≥50% forest cover, and we therefore used four fuel consumption measurements of pasture burning in Brazil, a fire type that often includes residual clearing [summarized in (*29*)]. Last, for forest fires, we used a single fuel consumption estimate from (*42*).

To convert cumulative FRP per area burned (megawatts per hectare) to the fuel consumption estimates, we used a q-q plot to match the distributions of observed fuel consumption from field measurements to FRP per area burned (fig. S14). First, both the distribution of observed fuel consumption (metric tons per hectare) and FRP per area burned (megawatts per hectare) were log-transformed to accommodate their nonlinear relationship and retain the long tail of both datasets. Second, a q-q plot was generated for each fire type (savanna, deforestation, and small clearing and agricultural fires), and their relationship was captured using a linear model. To remove the influence of occasional extremes in observed FRP, we used the 2nd to 98th percentile of FRP per area burned to fit the model and identify minimum and maximum fuel consumption. This resulted in a model mean, minimum, and maximum value close to the observations. The same approach could not be applied to forest fires because only a single fuel consumption measurement was available for this fire type (fig. S14, F and G). For forest fires, we directly translated the distribution of FRP per area burned into fuel consumption by matching the mean values for the Amazon biome. As this approach potentially results in more outliers, we used a more conservative 10th and 90th percentile threshold to cap minimum and maximum fuel consumption estimates.

Last, we translated per-fire estimates of dry matter burned to carbon emissions (fig. S15), using GFED4 (*13*) emissions factors (grams of species per kilogram of dry matter combusted): deforestation fires (491 g C per kg dry matter), forest fires (491 g C per kg dry matter), small clearing and agricultural fires (480 g C per kg dry matter), and savanna and grassland fires (488 g C per kg dry matter). Because deforestation and forest fires are not separated in GFED4s, we used the same emissions factors for both fire types. We note, however, that total carbon emissions per kilogram of dry matter burned are highly constant across land cover types, in contrast to emissions of the actual trace gasses (e.g., CO_2_, CO, CH_4_, etc.).

### 2019 analysis of variability in fire activity by fire type

To better understand the drivers of regional fire activity in forested systems, we explored time series of fire detections by fire type for the southern hemisphere Amazon and a larger study region (Fig. 3) as well as for Brazil and the five Brazilian states along the arc of deforestation (fig. S16). This analysis included deforestation fires, forest fires, and small and agricultural fires, the three fire types characterized by ≥50% tree cover (fig. S3). We compared results to long-term (2012–2018) average daily fire detections from Suomi NPP VIIRS using a 50% tree cover threshold at 0.005° resolution. To investigate the impact of climate on temporal variability, we calculated average daily rainfall from the Global Precipitation Measurement Integrated Multi-satellite Retrievals version 06 (*55*) across all 0.1° grid cells, the spatial resolution of precipitation data, with greater than 50 fire detections. Last, we investigate the relative contribution of long-duration fires, and we explore the relative contribution new fire starts, fires that burned 1 to 10 days, and fires that burned longer than 10 days to daily fire activity (Fig. 4 and fig. S17).

## Supplementary Materials

### This PDF file includes:

Supplementary Text Figs. S1 to S17 Tables S1 and S2 Data S1 and S2 References
