# Supplementary material for: Tracking and classifying Amazon fire events in near real time
Source: Sci Adv. 2022 Jul 29;8(30):eabd2713. doi: 10.1126/sciadv.abd2713 (PMC9337759; doi:10.1126/sciadv.abd2713)
Supplement: Supplementary file 1 — Supplementary Text Figs. S1 to S17 Tables S1 and S2 Data S1 and S2 References [file sciadv.abd2713_sm.pdf]

Science Advances
AAAS

Supplementary Materials for

Tracking and classifying Amazon fire events in near real time

Niels Andela *et al.*

Corresponding author: Email: Niels Andela, niels.andela@bezerocarbon.com

Sci. Adv. **8**, eabd2713 (2022)
DOI: 10.1126/sciadv.abd2713

This PDF file includes:

Supplementary Text
Figs. S1 to S17
Tables S1 and S2
Data S1 and S2
References

Supplementary Text

A comparison of fire type classification to PRODES deforestation data

To investigate the quality of our fire type classification, we first compared the 2019 fire classification results to 2019 deforestation estimates for Brazil (*6*). To enable near-real time data production, our algorithm only considers historic deforestation (2014-2018), and 2019 PRODES data therefore provide an independent reference. Here, we assume that fire events with ≥25% deforestation during 2019 are likely associated with deforestation. Of these 2019 deforestation fires, fire classification results stabilized over time (Fig. S6). On day one of a new fire start, most fires were classified as either small clearing or savanna fires, depending on fractional tree cover in the ignition locations. Persistent, multi-day fire activity is a key characteristic of deforestation and forest fires, and thus evidence for continued fire activity is needed to distinguish these fire types except in cases where 2019 deforestation fires also overlap with historic deforestation (2014-2018, see Fig. S3). By the second day, about 67% of active fire detections for all fires with ≥25% of grid cells overlapping with new deforestation during 2019 were accurately attributed to deforestation fires (Fig. S6). The classification accuracy of low and moderate confidence deforestation fires gradually increased over time, improving the overall accuracy of all confidence classes combined to near 80% of fire detections within one week. The major source of misclassification for these 2019 deforestation events was forest fires, especially for low confidence fire classifications, or for high confidence deforestation fires that grew large enough to be reclassified as forest fires based on size.

Using historic deforestation data in the classification scheme (see Fig. S3), 68% of active fire detections were accurately attributed to deforestation by the end of the season, or 56%, 79%, and 76% for low, moderate, and high confidence classes, respectively (Fig. S7). Most of the misclassified deforestation fire detections (22%) were identified as forest fires, with 6% as small clearing and agricultural fires and 3% as savanna or grassland fires. Without historic deforestation data as a guide, classification accuracies were somewhat lower, especially for high confidence fires: 64% of active fire detections were accurately attributed to deforestation, or 56%, 81%, and 65% for low, moderate, and high confidence classes. For the misclassified fire detections, 25% were assigned as forest fires, 8% as small clearing and agricultural fires and 3% as savanna or grassland fires. These results highlight the added value of historic deforestation data to rapidly identify fire activity from repeated deforestation fires and new deforestation fires in adjacent forest areas.

Of all fires classified as deforestation fires in the Brazilian Amazon, 74% had some spatial overlap with recent deforestation, as identified by PRODES (Fig. S8). This concurrence was highest for high-confidence deforestation fires (90%), with 49% and 51% overlap for moderate and low confidence fires, respectively. While misalignment between 2019 fire classification and PRODES data likely indicates errors of commission, especially for low and moderate confidence deforestation fires, two additional factors make it difficult to quantify the magnitude of commission errors in this study. First, PRODES does not map deforestation activity in secondary forests, a major component of new clearing activity in recent years (*56*). Second, PRODES only maps patches of forest loss larger than 6.25 hectares, and many smaller deforestation fires were assigned to lower confidence classes. Notably, there is some overlap between PRODES deforestation and fires classified as small clearing and agricultural fires in this study, as fires with very few active fire detections are challenging to classify accurately.

A small portion of the fire detections (11%) attributed to forest fires in this study originated from fires with ≥25% of grid cells overlapping with 2019 deforestation (Fig. S9). However, forest fires did partially overlap with both historic and 2019 deforestation. Large fires are likely to encompass several land cover types, and therefore some overlap with other classes is expected.

Considering all confidence levels, 31% of active fires attributed to forest fires occurred in fires with 10% or more overlap with 2014-2019 deforestation. However, the majority of this overlap with deforestation was confined to low (57%) and moderate (57%) confidence classes. Overall, low deforestation fractions in fires classified as forest fires suggests that most forest fires originated from other ignition sources, including abundant small clearing and agricultural fires across the region, rather than deforestation fires. However, a more detailed study of this relationship is warranted, based on the ignition time and locations for individual fire events in this study.

Evaluation of results compared to the Monitoring of the Andean Amazon Project (MAAP)

To provide an independent assessment of the accuracy of our model, we compared our fire type classification to 2,099 "major" fires identified by the MAAP project for the Brazilian Amazon and available for 2020 (*27*). To enable this comparison, we expanded our dataset to include 2020 based on the same methodology. MAAP classified fires that produce significant smoke into four distinct classes, "deforestation fires" associated with clear-felled vegetation for agricultural expansion, "forest fires", "cropland and pasture", and "grassland" based on expert visual interpretation of various satellite data products, including high-resolution (<4 m) commercial imagery. The comparison of our individual fire classification product to this subset of Amazon fire activity provides an initial estimate of errors of omission and commission but also highlight differences in fire type definitions (Fig. S10). Close agreement between MAAP and our dataset was found, with 75% of deforestation, 69% of grassland, and 64% of forest fires identified by MAAP accurately classified in this study, giving an approximate estimate of errors of omission. However, these results also reflect differences in the definitions. For example, 23% of MAAP grassland fires were classified as forest fire by our approach, and 15% of forest fires as savanna fires, possibly the result of different thresholds to separate forests and savannas. Our results also highlight the challenges associated with accurately separating forest and deforestation fires, with 22% of MAAP deforestation fires being misclassified as forest fire in our study. A lower percentage of active fire detections classified as deforestation (57%), savanna (18%), and forest (47%) in our study had the same classification in MAAP, highlighting larger errors of commission. However, these differences were to a large extent driven by the inclusion of a specific cropland and pasture fire class in MAAP, that had strong overlap with savanna fires (50%), but also with deforestation (31%) and forest fires (29%) in our study. The significant overlap of MAAP cropland and pasture fires with our deforestation fire class could be expected for two reasons. First, MAAP uses a two year-threshold to assign deforestation fires to recent clearing, while we included 5 years of historic data (Fig. S2). Second, pasture burning often includes some residual clearing and is therefore often characterized by higher fuel consumption (Fig. S14, *29*), when looking at the fire behavior this might appear similar to fires following earlier stages of deforestation. Similarly, many forest fires originate from burning in open cover types and burn into neighboring forest areas, and it is unclear how MAAP resolves these mixed fire types. In our approach, fire type is based on the fractional tree cover within the final burn perimeter, among other metrics. Overall, largely consistent results from the two approaches highlights the novelty of both products and the potential for these datasets to inform regional land management, conservation, and scientific advances regarding the nature and impacts of fire in the Amazon and surrounding biomes.

Fire type classification accuracy compared to Sentinel-2 image pairs

We assessed the accuracy at which we can separate deforestation from understory forest fires, the two main types of multi-day fires in the South American study region and an important distinction for accurate estimates of carbon emissions from deforestation and forest degradation (Table S2). Because of the class imbalance, we took a stratified random sample of 100 deforestation

and 100 forest fires across the South American study domain in 2019. We focused on fires that started in August, to avoid issues of cloud cover, and used pre- and post-fire Sentinel-2 images at 10 m resolution to interpret the reference fire type. In total, this resulted in a reference set of 194 fires across 118 image pairs after excluding 6 fires due to cloud cover (N=1) or inconclusive evidence of burning (N=5). Overall, the accuracy of fire type classification improved with fire size. The overall accuracy was 66% for fire events and 92% for active fire detections (large fires include more active fire detections; Table S2). The largest difference in accuracy was observed for forest fires, with 55% User's accuracy (reflecting commission error) for fire events increasing to 93% User's accuracy for fire detections, likely indicating a more skewed distribution of fire size and associated fire detections compared to deforestation fires. Deforestation fire classification was more stable, with 78% User's and 63% Producer's accuracy for fire events, and 87% User's and 71% Producer's accuracy for fire detections.

Algorithm accuracy in near-real time

To better understand the algorithm performance in near-real time, we compared our near-real time results to the end of year classification. The long duration of most deforestation, forest, and savanna fires increases the accuracy of the near-real time fire type classification (Fig. 4 and S17). Even though it takes time to accurately separate deforestation and forest fires from other fire types (e.g., Fig. S6), individual fires often burn for weeks or months. As a result, during the peak of the burning season (August and September), on average 86% of all fire detections were associated with fires that ignited on a previous day (Fig. 4), compared to only 14% of all fire detections from new fire starts. Thus, the ability to attribute fire detections to specific fire types on any given day reflects the weighted average of the classification accuracy of multi-day fires (days 2+, 86%) and the classification accuracy of new fire starts (day 1, 14%). Using the end-of-season classification as the reference, about 76% of active fire detections from deforestation fires were correctly classified on the same day during August and September, increasing to 92% with a one-week lag time. Forest fire classification improved markedly over the season, based on the growing fraction of active fire detections from long-duration fires. Attribution of small clearing and agricultural fires was consistent throughout the 2019 fire season, since most start in this class at initial detection.

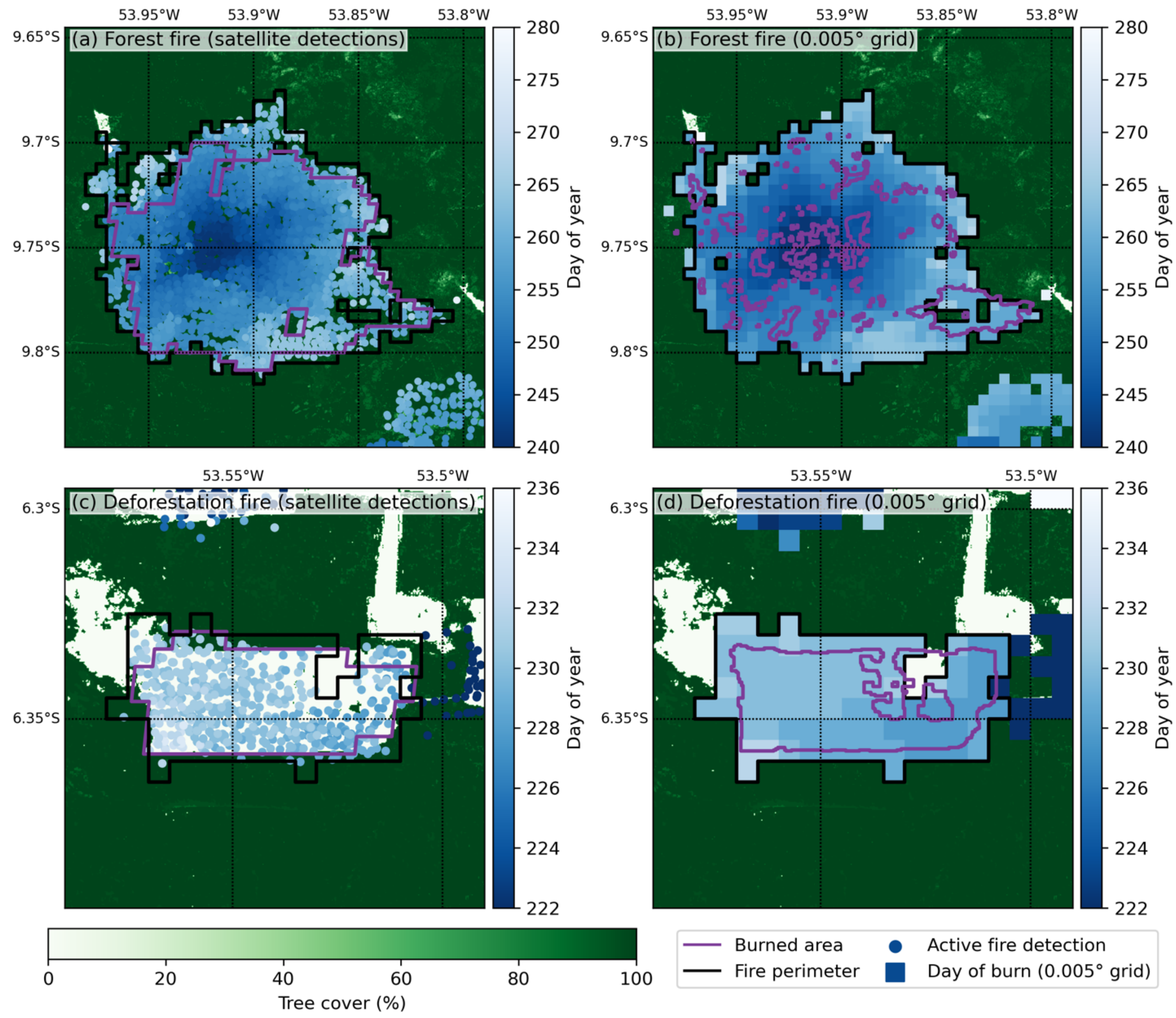

Fig. S1: The spatial and temporal evolution of VIIRS active fire detections for Amazon deforestation and forest fires provides an estimate of daily burned area needed for the Global Fire Atlas algorithm. Day of active fire detections (circles) from both VIIRS instruments for (a) a large forest fire and (c) a deforestation fire in Brazil. Panels (b) and (d) show the estimated daily fire progression on a 0.005º (~550 m) grid. The background depicts fractional tree cover (*10*). Black lines indicate the fire perimeters derived from the VIIRS active fire detections and purple lines show the corresponding burned area estimates from the MCD64A1 C6 product (*28*) in (a) and (c) and Landsat-derived burned area estimates from MapBiomas in (b) and (d).

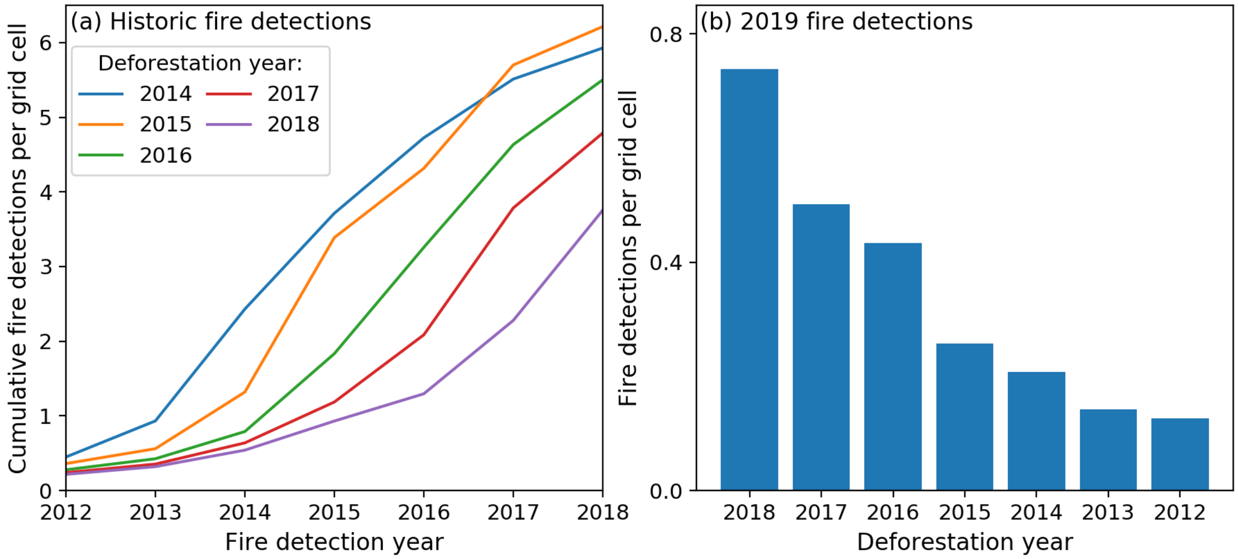

Fig. S2: Elevated fire activity across deforested grid cells was observed up to five years after the year of initial deforestation detection by PRODES. (a) Cumulative VIIRS fire detections per 550 m grid cell in comparison to the year of deforestation detection. (b) Density of 2019 VIIRS fire detections for grid cells with different years of historic deforestation. All data are for Brazil and based on active fire detections from the VIIRS instrument onboard Suomi-NPP.

Table S1: Selected training data for forest fires.

Region	Fire number	Average fire detections	Average fire radiative power (MW)	Average fire size (km^2)
Inside the Amazon	28	693	13.1	56.2
Outside the Amazon	49	3289	19.9	283.1
Total	77	2345	17.4	200.6

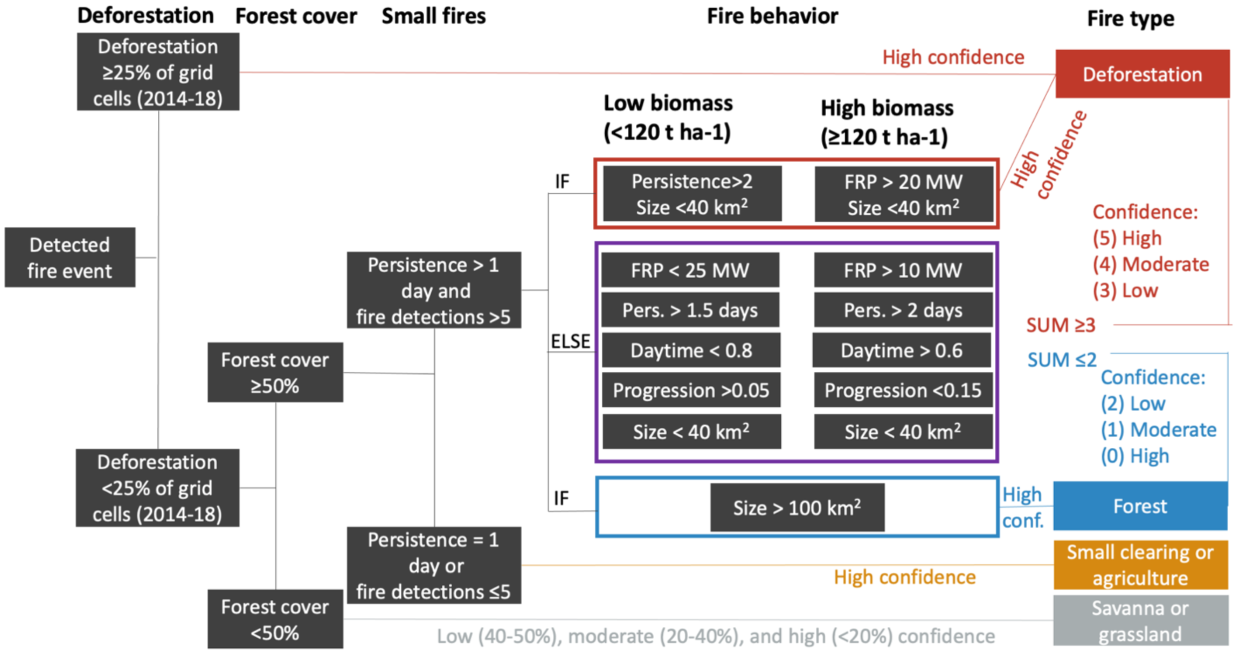

Fig. S3: Flow diagram of the fire type classification system and confidence level assessment. Fires were separated into deforestation, forest, small clearing and agricultural, and savanna and grassland fires using metrics of fire behavior and land cover information. The initial separation between high-confidence deforestation fires and savanna and grassland fires uses historic deforestation data (2014 – 2018) and fractional tree cover (2014). For all remaining fires with ≥50% tree cover, we first isolate small clearing and agricultural fires based on low fire persistence and number of fire detections. To further separate deforestation fires from forest fires we use a separate classification for low biomass (left column, <120 t ha^{-1}) and high biomass (right column, ≥120 t ha^{-1}) systems based on observed differences in fire behavior in moist and dry forests, respectively (see Fig. S4).

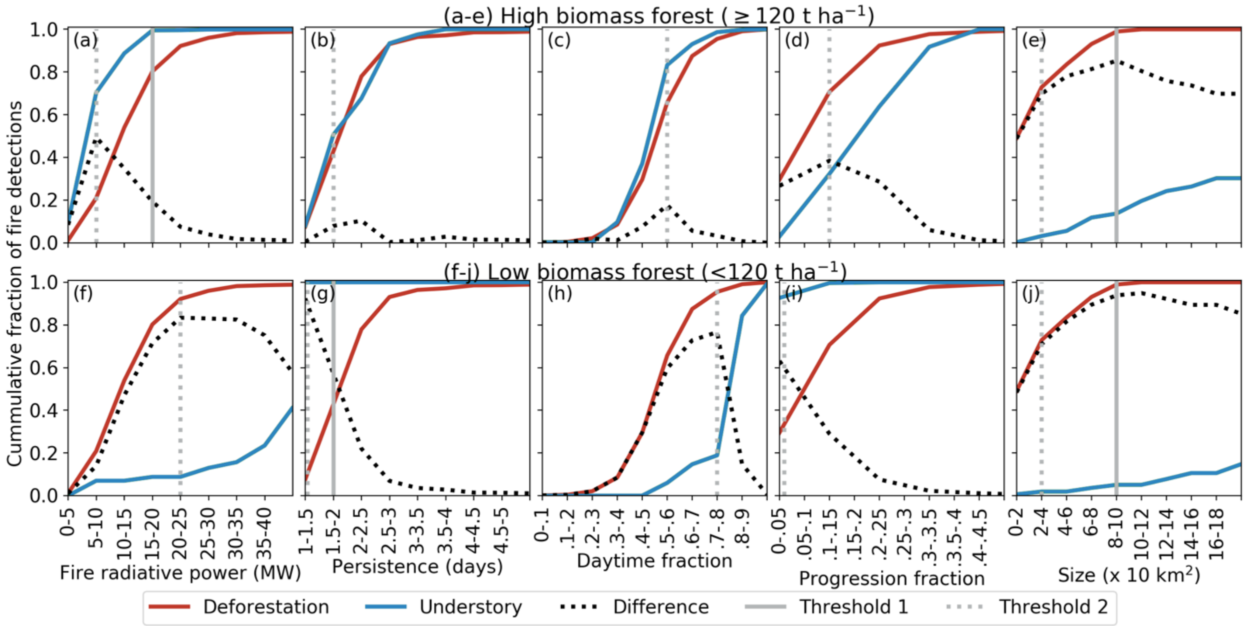

Fig. S4: Five fire behavior metrics distinguish deforestation from understory forest fires. (a) Fire radiative power is a measure of instantaneous energy release by a fire. (b) Persistence indicates the number of days a fire was active within any given 550 m grid cell. (c) The daytime fraction shows the ratio of 1:30 PM to 1:30 AM active fire detections, indicative of the magnitude of diurnal variability in fire behavior for multi-day fires. (d) The progression fraction is the fraction of FRP observed on the second day of the fire compared to the sum of observed FRP on the first two days. Gradual fire spread, typical of slow-moving forest fires in high biomass ecosystems, results in more equal FRP on days one and two, while the progression from flaming to smoldering combustion in deforestation fires results in a rapid decline in FRP. (e) Fire event size. Figures (f-j) are like (a-e) but for fires in forested ecosystems with average biomass below 120 t ha^{-1}. Threshold 1 (solid gray lines) indicates high confidence classification thresholds shown in Fig. S3 and threshold 2 (dashed gray lines) indicate the five thresholds used in the multivariate decision scheme shown in Fig. S3 to assign fire type and associated confidence levels.

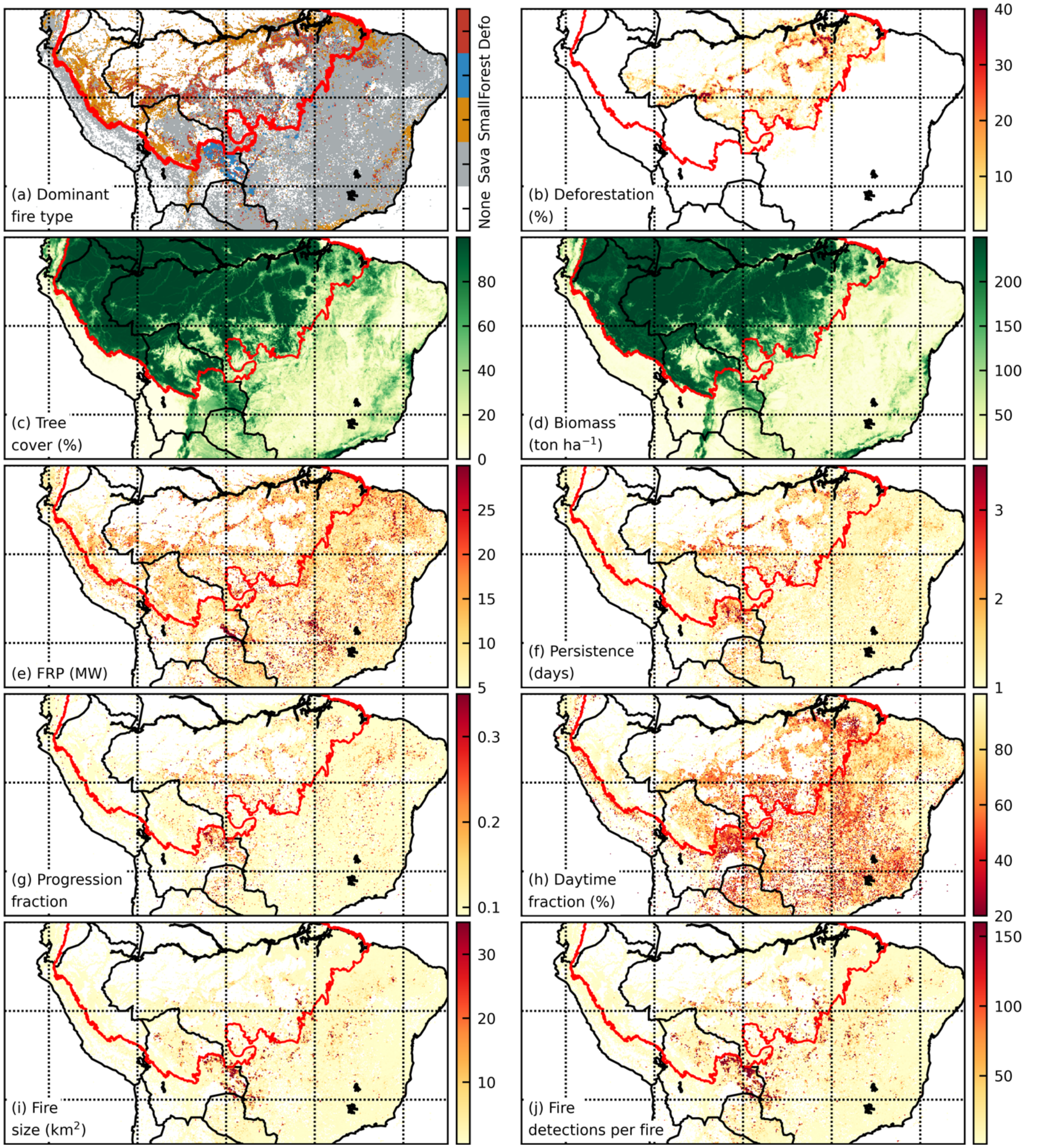

Fig. S5: Spatial patterns of the land cover data and individual fire metrics used to separate among deforestation, forest, small clearing and agriculture, and savanna and grassland fire types. (a) dominant fire type, (b) deforestation fraction during 2014 – 2018 (*6*), (c) Tree cover fraction in 2014 (*10*), (d) aboveground live biomass (*44*), (e) fire radiative power (FRP), (f) fire persistence, (g) progression fraction, (h) daytime detection fraction, (i) fire size, and (j) the number of fire detections per fire event. All figure panels are at 0.1° resolution using fire data from April – December 2019. Subplot (a) shows the dominant fire type based on fire detections, subplots (b-h) show average values derived from higher resolution 0.005° gridded data, and subplots (i-j) show averages weighted by individual fire number.

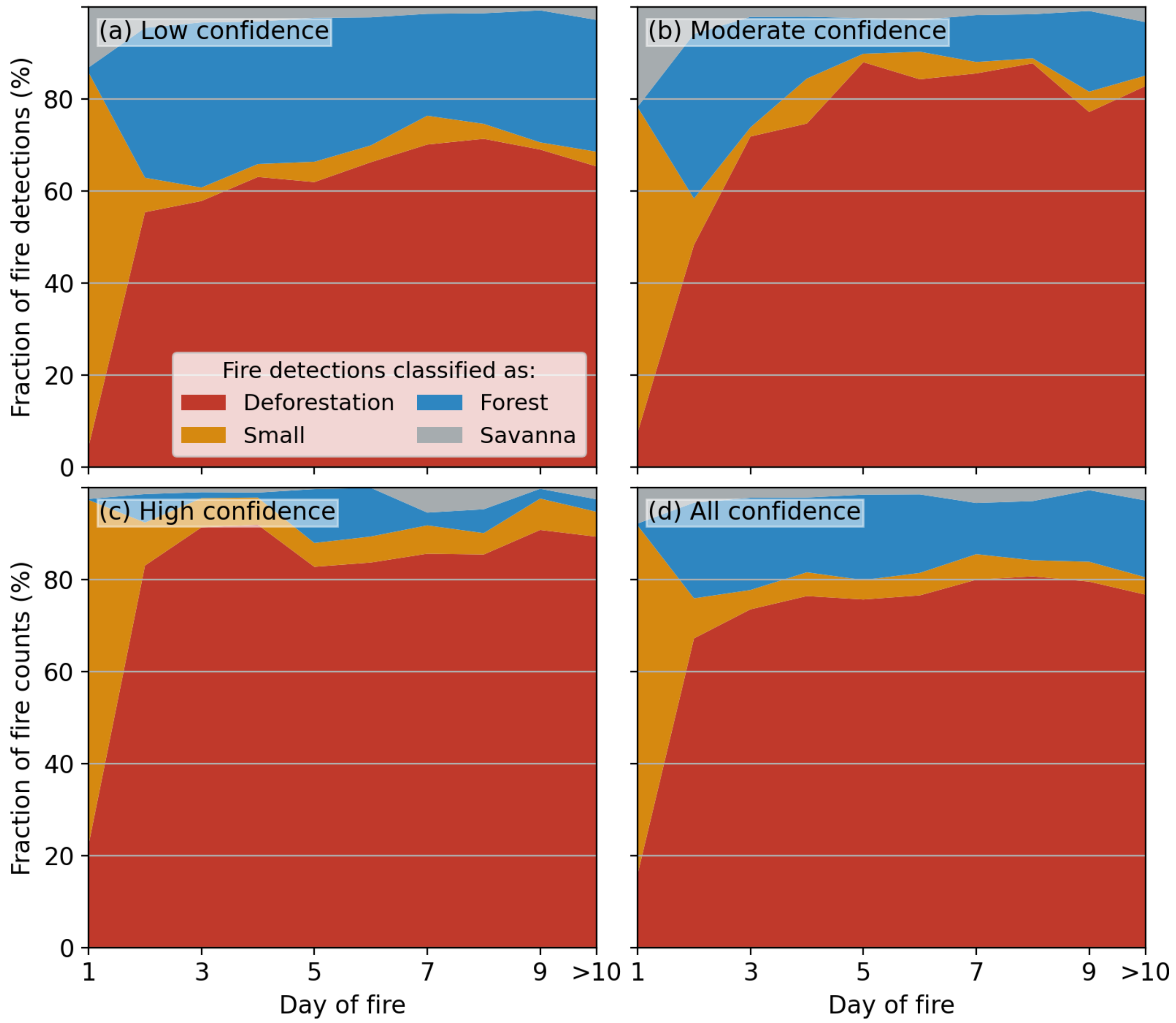

Fig. S6: Evolution of fire type classification after the start of each fire (day=1) for all fires with ≥25% overlap with 2019 deforestation from PRODES (*6*)**.** (a) Low confidence fires, (b) moderate confidence fires, (c) high confidence fires, (d) all confidence classes. The start day (day 1) of each fire was defined as the 1st percentile of the day of year of all fire detections within the fire perimeter.

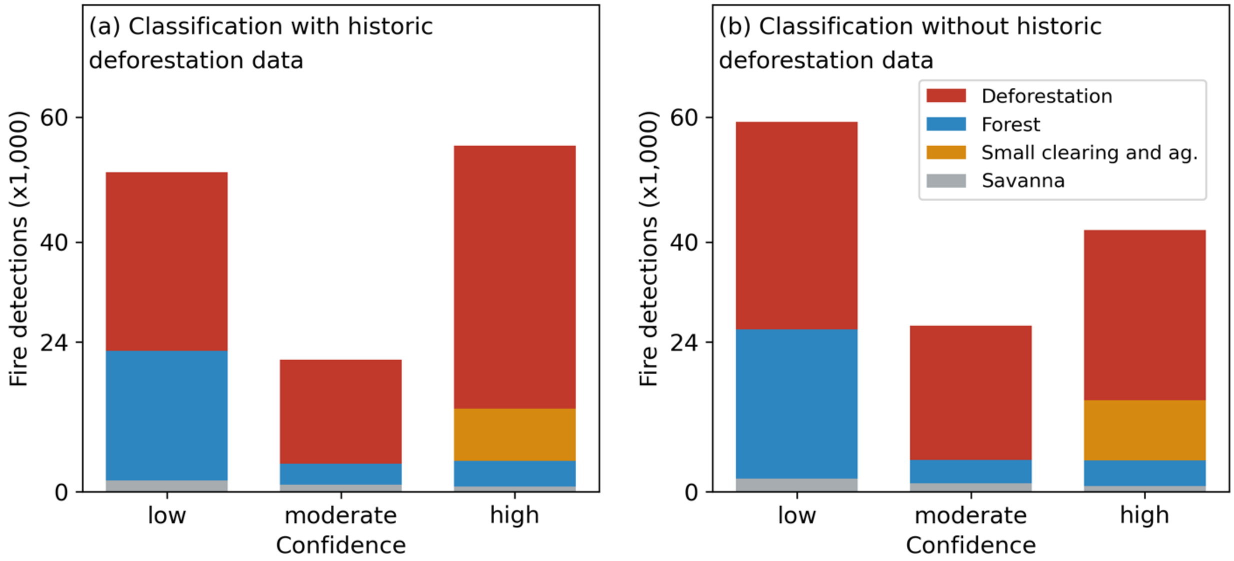

Fig. S7: Classification of 2019 deforestation fires with and without information about historic deforestation (2014-2018). (a) Classification of all fire events with ≥25% of grid cells overlapping with 2019 deforestation using our full approach, including historic deforestation data (Fig. S3). (b) Classification of 2019 deforestation fire events without using historic deforestation data.

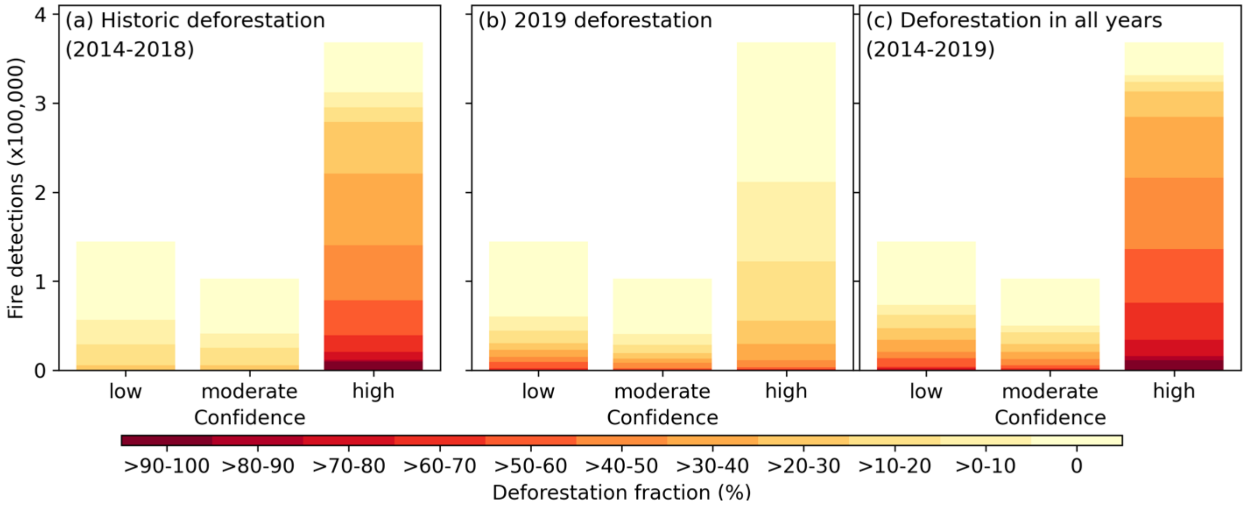

Fig. S8: Deforestation fraction of all fires classified as deforestation fires in our analysis, weighted by fire detections. (a) Historic deforestation (2014-2018), (b) preliminary 2019 deforestation estimates, and (c) deforestation in all years (2014-2019). Data are for Brazil only, based on deforestation estimates from PRODES (*6*).

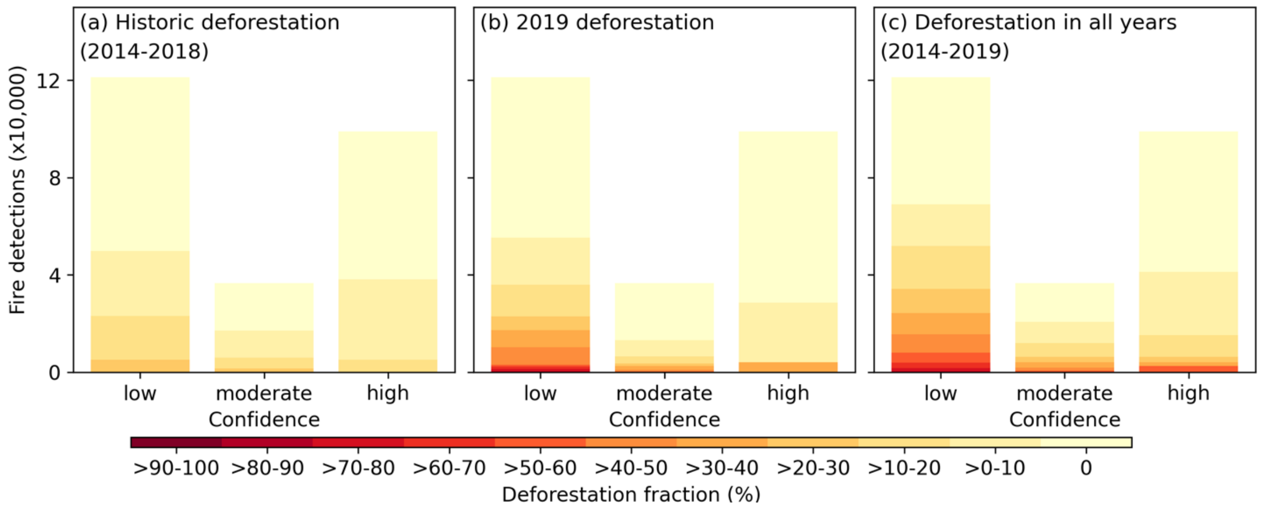

Fig. S9: Deforestation fraction of all fires classified as forest fires in our analysis, weighted by fire detections. (a) Historic deforestation (2014-2018), (b) 2019 deforestation estimates, and (c) deforestation in all years (2014-2019). Data are for Brazil only, based on deforestation estimates from PRODES (*6*).

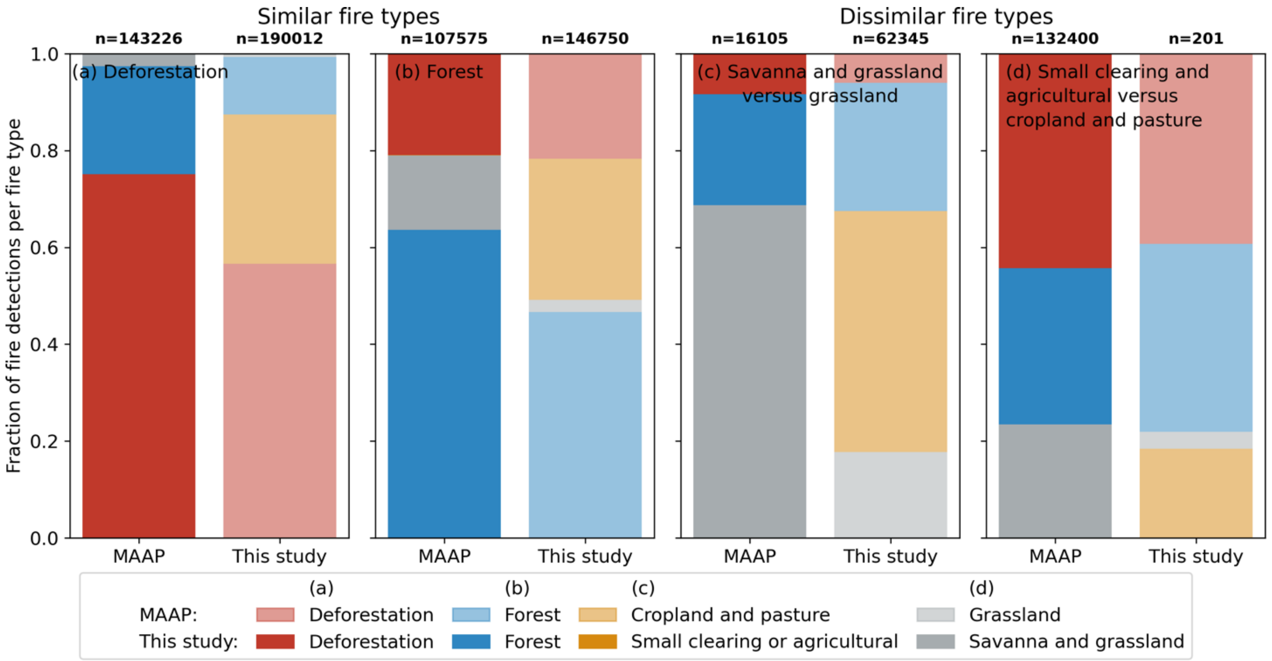

Fig. S10: Comparison of fire types defined here to similar and more dissimilar fire types identified by MAAP for 2020 in Brazil. In (a), the left bar includes all fire detections from fires classified as deforestation fires by MAAP and highlights the corresponding fire type from our study (dark colors), the right bar indicates all fires classified as deforestation fire in our study and highlights the corresponding fire type from the MAAP database (light colors). (b) is like, (a) but for forest fires. (c) and (d) are like (a) but for fire types that are less similar. The "n" value above each figure indicates the total number of active fire detections in each bar. Differences in classification in the left bars can be interpreted as errors of omission and the right bars as errors of commission of our dataset compared to MAAP. However, these "errors" are partly driven by differences in the definition of the fire types (see Supplementary text for further discussion).

Table S2: The algorithm accurately separated most deforestation from understory forest fires. (a) Accuracy assessment of fire events and (b) accuracy assessment of active fire detections.

a) Fire events		Reference data			
		Deforestation	Forest	Total	User's Accuracy
Classification data	Deforestation	75	21	96	78%
	Forest	44	54	98	55%
	Total	119	75	194	
	Producer's accuracy	63%	72%		
	Overall Accuracy = 66%				

b) Fire detections		Reference data			
		Deforestation	Forest	Total	User's Accuracy
Classification data	Deforestation	4,309	667	4,976	87%
	Forest	1,721	22,997	24,718	93%
	Total	6,030	23,664	29,694	
	Producer's accuracy	71%	87%		
	Overall Accuracy = 92%				

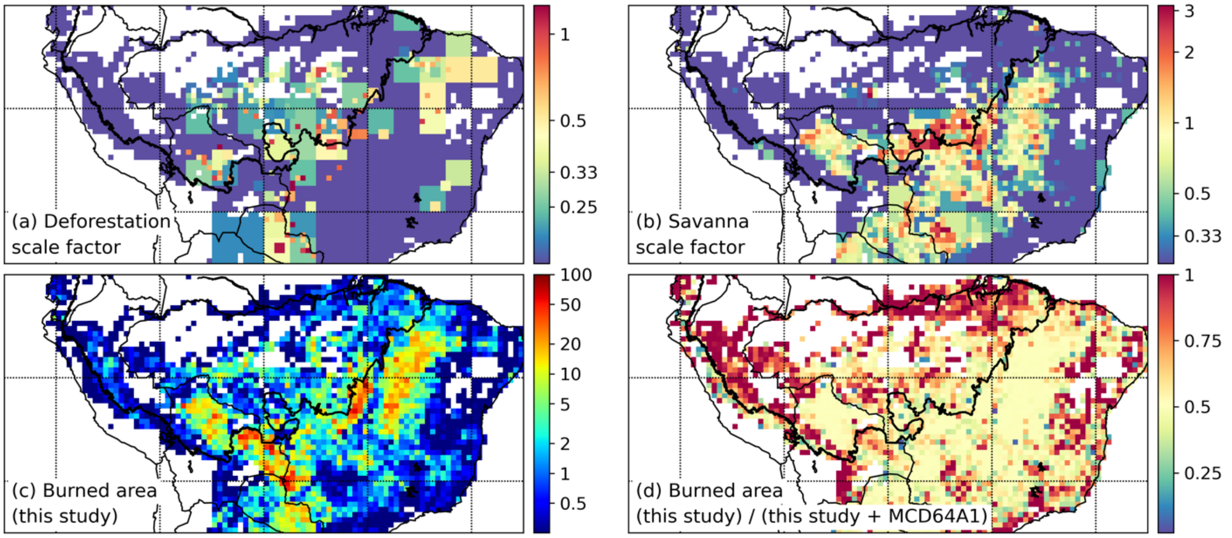

Fig. S11: Scaling approach to convert fire perimeters from active fires to estimated burned area by fire type. We used two separate scaling factors for (a) deforestation and (b) savanna fires to match burned area within fire perimeters derived here to MODIS collection 6 burned area estimates (*28*). Fixed scaling factors were used for small clearing and agricultural fires and forest fires (see methods). (c) shows total estimated burned area from all fire types combined and (d) shows the relative contribution of burned area estimated here to the sum of MCD64A1 and burned area from this study. In (d), a value of 0.5 indicates equal burned area between both datasets, with observed deviation originating from burned area estimates in the small clearing and agricultural fires and forest fire classes that were not matched to MCD64A1. All subplots are shown at 0.5° resolution and grid cells with less than 1 km^2 burned area in (c) are masked white to aid interpretation.

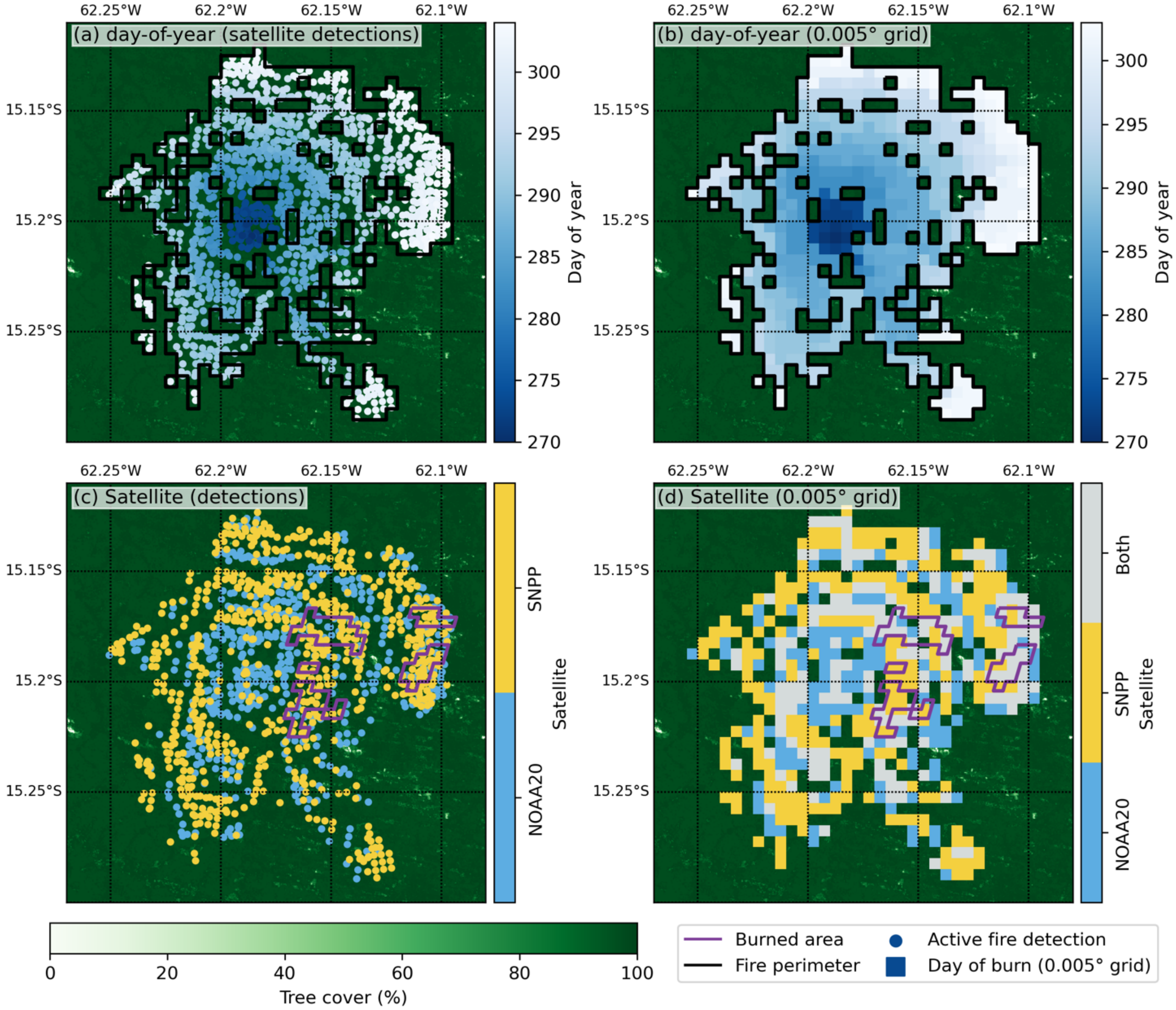

Fig. S12: Frequent near-nadir observations from the combined VIIRS sensors onboard Suomi-NPP and NOAA20 result in unprecedented capability to track forest fire expansion under dense forest canopy. (a) Active fire detections by day-of-burn, (b) gridded day-of-burn at 0.005° (550 m) resolution, (c) active fire detections by instrument, and (d) grid cells identified as burned by either VIIRS onboard Suomi-NPP, NOAA20 or both VIIRS sensors. Black lines indicate the fire perimeters from clustering VIIRS active fire detections into individual events (top panels), purple lines show the extent of MODIS burned area data (bottom panel, *28*).

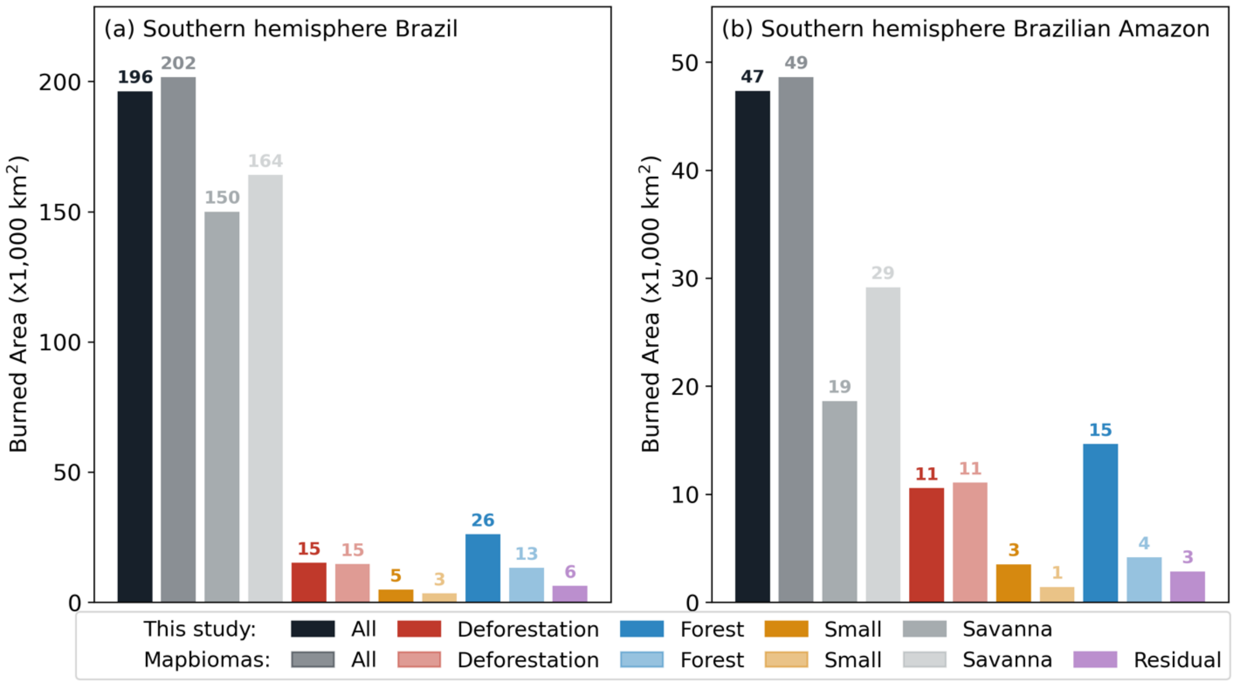

Fig. S13: Close comparison was found between regional burned area estimates from our approach and burned area from MapBiomas for most fire types. (a) Burned area estimates for southern hemisphere Brazil (0-25°S), and (b) burned area estimates for the southern-hemisphere Brazilian Amazon.

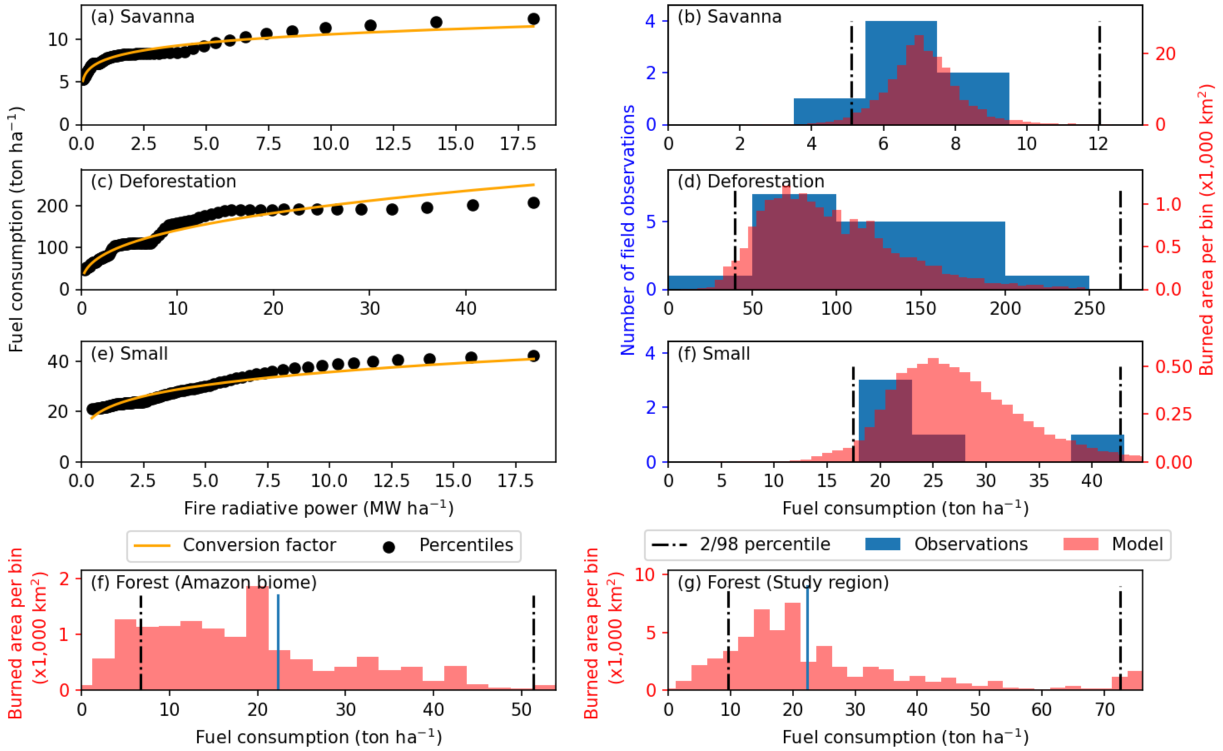

Fig. S14: Distribution of observed and modeled fuel consumption estimates. (a) Savanna conversion factors between cumulative fire radiative power per area burned (MW ha^{-1}) and fuel consumption per area burned (ton dry matter ha^{-1}) were derived using a q-q plot. (b) Distribution of fuel consumption from savanna experimental burns (blue) and modeled savanna burned area per fuel consumption bin. (c) and (d) are like (a) and (b) but for Deforestation fires and (e) and (f) are like (a) and (b) but for small clearing and agricultural fires. (f) and (g) show modeled fuel consumption compared to the single field measurement available for fuel consumption in tropical forests. To avoid possible outliers in modeled fuel consumption estimates we used the 2nd and 98th percentile (10th and 90th percentile for forest fires) of observed fire radiative power per area burned as minimum and maximum fuel consumption estimates (intermittent black lines). Both mean, min, and max fuel consumption from our approach are in close agreement with the field observations.

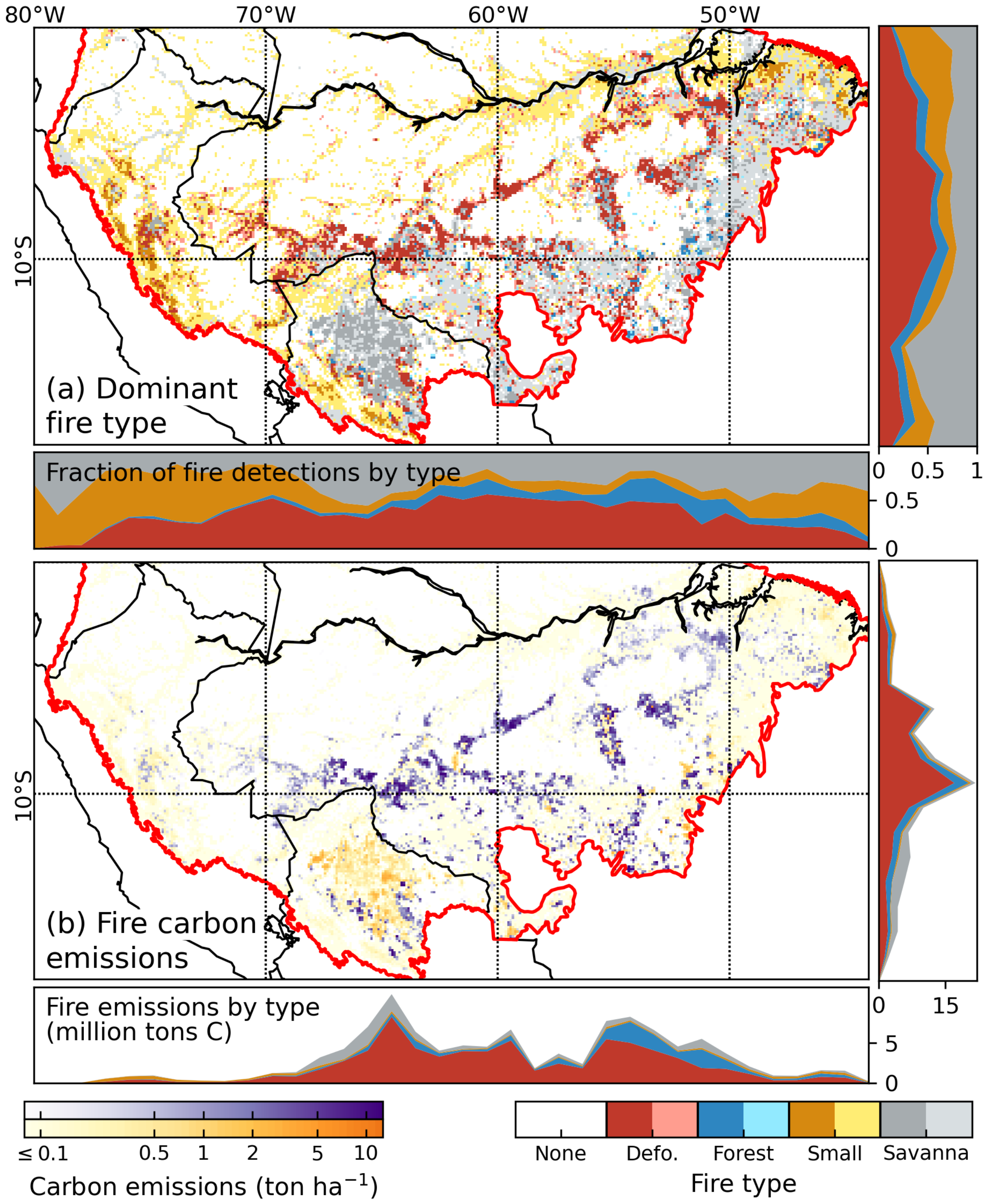

Fig. S15: Separation of deforestation and forest fires from other fire types identifies regions with long-term carbon losses from fire. (a) Dominant fire type and (b) estimated carbon emissions during April – December 2019 at 0.1° resolution. In (a), grid cells with fewer than 50 fire detections are shown in lighter shades of the same color. In (b), long-term carbon losses from deforestation and forest fires are shown in purple; short-term carbon losses from small clearing and agricultural fires and savanna and grassland fires are shown in orange. Insets show the contribution of different fire types per 1° bin of latitude and longitude (See Fig. 2 for the full South American study domain).

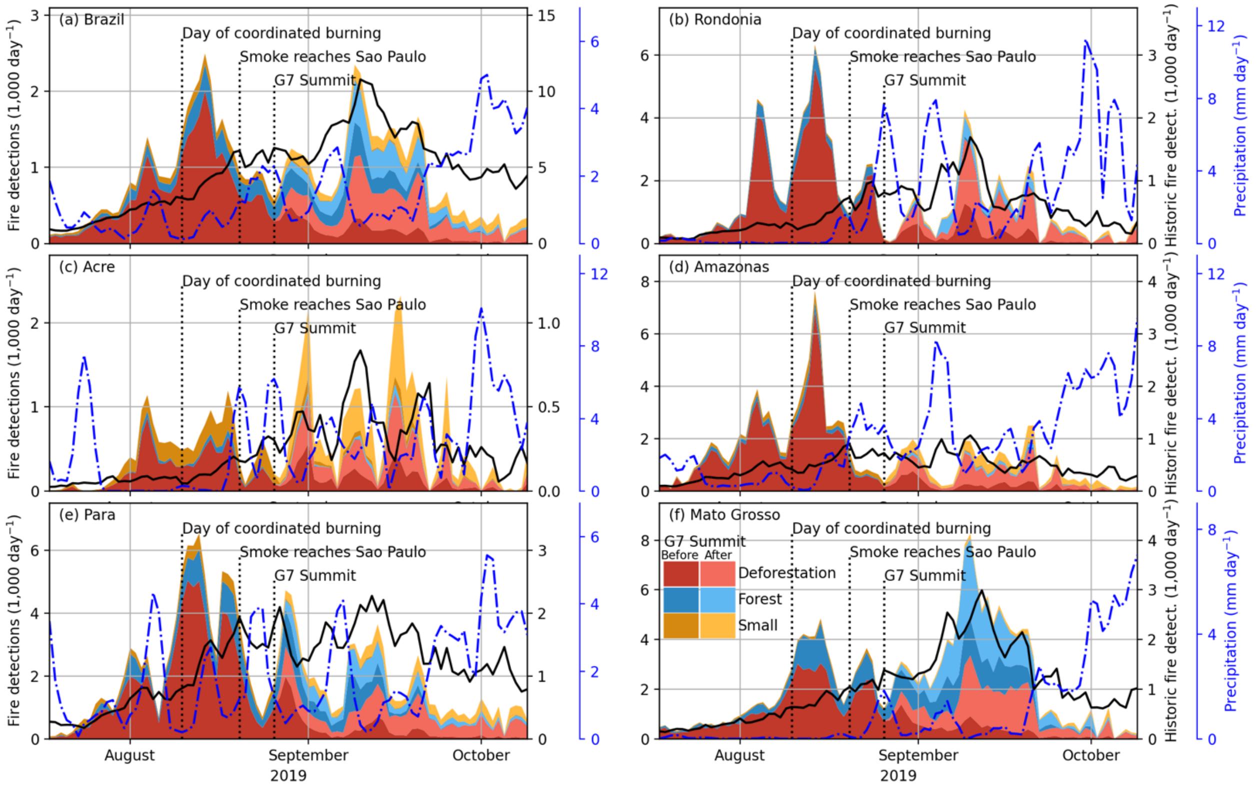

Fig. S16: Breakdown of fire detections by fire type for Brazil and five Brazilian states along the arc of deforestation. Light shades highlight the contribution from new fires started after the G7 Summit on August 24-26th. The solid black lines (right y-axis) indicate historic average (2012-2018) daily fire detections in forested systems (≥50% tree cover) based on a single VIIRS sensor (Suomi NPP). The intermittent blue lines (second right y-axis) indicate average daily precipitation across all 0.1° grid cells with greater than 50 fire detections.

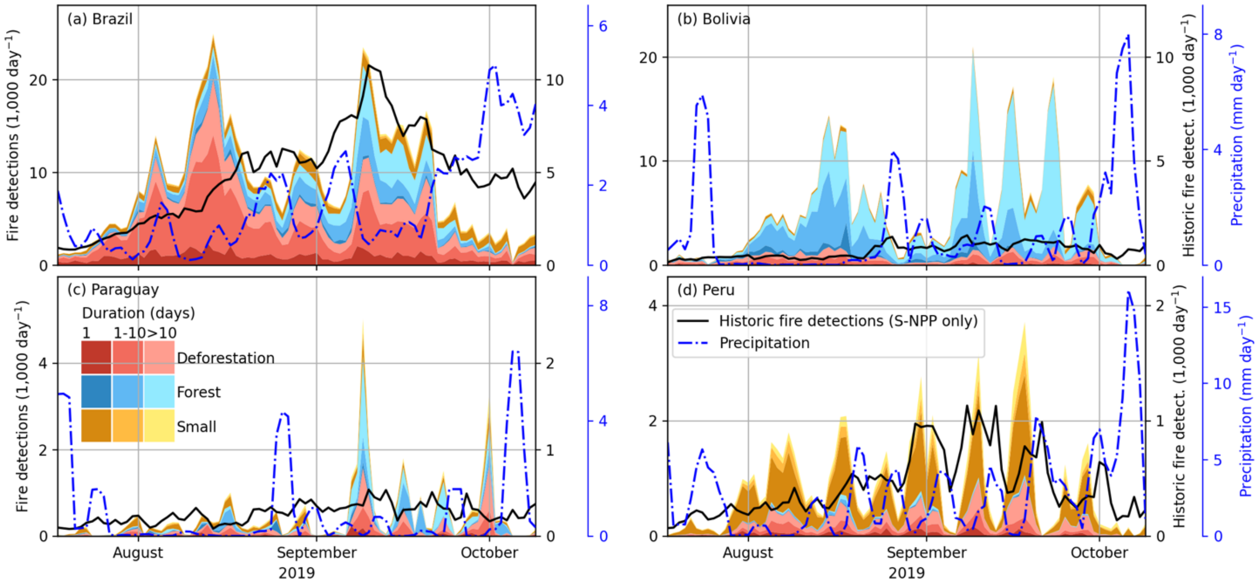

Fig. S17: Regional breakdown of fire detections by fire type and duration. Dark shades indicate the contribution from new fire starts, and lighter shades from fires that burned between 1 and 10 days or longer than ten days, respectively. The solid black lines (right y-axis) indicate historic average (2012-2018) daily fire detections in forested systems (≥50% tree cover) based on a single VIIRS sensor (Suomi NPP). The intermittent blue lines (second right y-axis) indicate average daily precipitation across all 0.1° grid cells with greater than 50 fire counts.

Supplementary Data for this manuscript

Data files S1 and S2 for 2019 and 2020 are archived alongside this manuscript (https://doi.org/10.5281/zenodo.6641625) and data are provided in near real time at www.globalfiredata.org.

Data S1: Two shapefiles of individual fire events by fire type for Southern Hemisphere South America (0–25°S). The first shapefile includes data from April – December 2019 and the second for 2020.

Data S1: Explanation of shapefile attribute table per fire event.

Attribute class	Attribute	Explanation / units
Fire type classification	Fire type	(1) savanna and grassland, (2) small clearing and agriculture, (3) forest, and (4) deforestation fires
	Confidence	(1) low, (2) moderate, and (3) high
Fire Atlas	Size	Fire size in km^2
	Start day	Day of new fire start as day of year (1-366)
	Duration	Fire duration in days
	Emissions	Fire carbon emissions (ton C)
Fire characterization	Tree cover	Average tree cover fraction within perimeter (%)
	Biomass	Average biomass within fire perimeter (ton ha^{-1})
	Deforestation	Fraction of 550 m grid cells with historic deforestation (2014 – 2018) within fire perimeter (%)
	FRP	Average fire radiative power (FRP) for all fire detections within fire perimeter (MW)
	Persistence	Average fire persistence across 550 m grid cells within fire perimeter (days)
	Progression	Average fire progression fraction across 550 m grid cells within perimeter (%)
	Daytime	Fraction of 1:30 pm detections (%) for all fire detections within fire perimeter
	Detections	Total active fire detections within fire perimeter

Data S2: Two shapefiles of satellite fire detections by fire type for Southern Hemisphere South America (0–25°S). The first shapefile includes data from April – December 2019 and the second for 2020.

Data S2: Explanation of shapefile attribute table per fire detection.

Attribute class	Attribute	Explanation / units
VIIRS fire detections	FRP	Fire radiative power (MW)
	DOY	Day of year (1-366)
Fire type classification	Fire type	(1) savanna and grassland, (2) small clearing and agriculture, (3) forest, and (4) deforestation fires
	Confidence	(1) low, (2) moderate, and (3) high

REFERENCES AND NOTES

1. Y. Malhi, J. T. Roberts, R. A. Betts, T. J. Killeen, W. Li, C. A. Nobre, Climate change, deforestation, and the fate of the amazon. *Science* **319**, 169–172 (2008).

2. J. Liu, K. W. Bowman, D. S. Schimel, N. C. Parazoo, Z. Jiang, M. Lee, A. A. Bloom, D. Wunch, C. Frankenberg, Y. Sun, C. W. O'Dell, K. R. Gurney, D. Menemenlis, M. Gierach, D. Crisp, A. Eldering, Contrasting carbon cycle responses of the tropical continents to the 2015–2016 El Niño. *Science* **358**, eaam5690 (2017).

3. J. Barlow, G. D. Lennox, J. Ferreira, E. Berenguer, A. C. Lees, R. M. Nally, J.R. Thomson, S. F. B. Ferraz, J. Louzada, V. H. F. Oliveira, L. Parry, R. R. de C. Solar, I. C. G. Vieira, L. E. O. C. Aragão, R. A. Begotti, R. F. Braga, T. M. Cardoso, R.C. de Oliveira Jr., C. M. Souza Jr., N. G. Moura, S. S. Nunes, J. V. Siqueira, R. Pardini, J. M. Silveira, F. Z. Vaz-de-Mello, R.C.S. Veiga, A. Venturieri, T. A. Gardner, Anthropogenic disturbance in tropical forests can double biodiversity loss from deforestation. *Nature* **535**, 144–147 (2016).

4. D. Skole, C. Tucker, Tropical deforestation and habitat fragmentation in the amazon: Satellite data from 1978 to 1988. *Science* **260**, 1905–1910 (1993).

5. R. S. De Fries, D. C. Morton, G. R. van der Werf, L. Giglio, G. J. Collatz, J. T. Randerson, R. A. Houghton, P. K. Kasibhatla, Y. Shimabukuro, Fire-related carbon emissions from land use transitions in southern Amazonia. *Geophys. Res. Lett.* **35**, L22705 (2008).

6. National Institute of Space Research (INPE), PRODES deforestation (2019); http://terrabrasilis.dpi.inpe.br/en/home-page/ [accessed 20 March 2020].

7. L. O. Anderson, Y. E. Shimabukuro, R. S. Defries, D. Morton, Assessment of deforestation in near real time over the brazilian amazon using multitemporal fraction images derived from Terra MODIS. *IEEE Geosci. Remote Sens. Lett.* **2**, 315–318 (2005).

8. B. Soares-Filho, R. Rajão, M. Macedo, A. Carneiro, W. Costa, M. Coe, H. Rodrigues, A. Alencar, Cracking Brazil's forest code. *Science* **344**, 363–364 (2014).

9. M. N. Macedo, R. S. DeFries, D. C. Morton, C. M. Stickler, G. L. Galford, Y. E. Shimabukuro, Decoupling of deforestation and soy production in the southern Amazon during the late 2000s. *Proc. Natl. Acad. Sci. U.S.A.* **109**, 1341–1346 (2012).

10. M. C. Hansen, P. V. Potapov, R. Moore, M. Hancher, S. A. Turubanova, A. Tyukavina, D. Thau, S. V. Stehman, S. J. Goetz, T. R. Loveland, A. Kommareddy, A. Egorov, L. Chini, C. O. Justice, J. R. G. Townshend, High-resolution global maps of 21st-century forest cover change. *Science* **342**, 850–853 (2013).

11. J. Barlow, Clarifying Amazonia's burning crisis. *Glob. Chang. Biol.* **26**, 319–321 (2020).

12. D. C. Morton, R. S. Defries, J. T. Randerson, L. Giglio, W. Schroeder, G. R. van der Werf, Agricultural intensification increases deforestation fire activity in Amazonia. *Glob. Chang. Biol.* **14**, 2262–2275 (2008).

13. G. R. van der Werf, J. T. Randerson, L. Giglio, T. T. van Leeuwen, Y. Chen, B. M. Rogers, M. Mu, M. J. E. van Marle, D. C. Morton, G. J. Collatz, R. J. Yokelson, P. S. Kasibhatla, Global fire emissions estimates during 1997–2016. *Earth Syst. Sci. Data.* **9**, 697–720 (2017).

14. D. I. Rappaport, D. C. Morton, M. Longo, M. Keller, R. Dubayah, M. N. dos-Santos, Quantifying long-term changes in carbon stocks and forest structure from Amazon forest degradation. *Environ. Res. Lett.* **13**, 065013 (2018).

15. P. M. Brando, J. K. Balch, D. C. Nepstad, D. C. Morton, F. E. Putz, M. T. Coe, D. Silvério, M. N. Macedo, E. A. Davidson, C. C. Nóbrega, A. Alencar, B. S. Soares-Filho, Abrupt increases in Amazonian tree mortality due to drought-fire interactions. *Proc. Natl. Acad. Sci. U.S.A.* **111**, 6347–6352 (2014).

16. A. C. Staver, P. M. Brando, J. Barlow, D. C. Morton, C. E. T. Paine, Y. Malhi, A. A. Murakami, J. del Pasquel, Thinner bark increases sensitivity of wetter Amazonian tropical forests to fire. *Ecol. Lett.* **23**, 99–106 (2019).

17. P. M. Brando, B. S.-Filho, L. Rodrigues, A. Assunção, D. Morton, D. Tuchschneider, E. C. M. Fernandes, M. N. Macedo, U. Oliveira, M. T. Coe, The gathering firestorm in southern Amazonia. *Sci. Adv.* **6**, eaay1632 (2020).

18. L. E. O. C. Aragão, L. O. Anderson, M. G. Fonseca, T. M. Rosan, L. B. Vedovato, F. H. Wagner, C. V. J. Silva, C. H. L. S. Junior, E. Arai, A. P. Aguiar, J. Barlow, E. Berenguer, M. N. Deeter, L. G. Domingues, L. Gatti, M. Gloor, Y. Malhi, J. A. Marengo, J. B. Miller, O. L. Phillips, S. Saatchi, 21st Century drought-related fires counteract the decline of Amazon deforestation carbon emissions. *Nat. Commun.* **9**, 536 (2018).

19. D. C. Morton, Y. Le Page, R. DeFries, G. J. Collatz, G. C. Hurtt, Understorey fire frequency and the fate of burned forests in southern Amazonia. *Philos. Trans. R. Soc. B Biol. Sci.* **368**, 20120163 (2013).

20. J. T. Randerson, Y. Chen, G. R. Van Der Werf, B. M. Rogers, D. C. Morton, Global burned area and biomass burning emissions from small fires. *J. Geophys. Res. G Biogeosci.* **117**, G04012 (2012).

21. W. J. Bond, Ancient grasslands at Risk. *Science* **351**, 120–122 (2016).

22. L. Giglio, W. Schroeder, C. O. Justice, The collection 6 MODIS active fire detection algorithm and fire products. *Remote Sens. Environ.* **178**, 31–41 (2016).

23. A. Cardil, S. de-Miguel, C. A. Silva, P. B. Reich, D. Calkin, P. H. S. Brancalion, A. C. Vibrans, J. G. P. Gamarra, M. Zhou, B. C. Pijanowski, C. Hui, T. W. Crowther, B. Herault, D. Piotto, C. Salas-Eljatib, E. N. Broadbent, A. M. A. Zambrano, N. Picard, L. E. O. C. Aragao, J.-F. Bastin, D. Routh, J. van den Hoogen, P. L. Peri, J. Liang, Recent deforestation drove the spike in Amazonian fires. *Environ. Res. Lett.* **15**, 121003 (2019).

24. D. C. Morton, R. S. DeFries, J. Nagol, C. M. Souza Jr., E. S. Kasischke, G. C. Hurtt, R. Dubayah, Mapping canopy damage from understory fires in Amazon forests using annual time series of Landsat and MODIS data. *Remote Sens. Environ.* **115**, 1706–1720 (2011).

25. N. Andela, D.C. Morton, L. Giglio, R. Paugam, Y. Chen, S. Hantson, G. R. van der Werf, J. T. Randerson, The Global Fire Atlas of individual fire size, duration, speed, and direction. *Earth Syst. Sci. Data* **11**, 529–552 (2019).

26. W. Schroeder, P. Oliva, L. Giglio, I. A. Csiszar, The New VIIRS 375 m active fire detection data product: Algorithm description and initial assessment. *Remote Sens. Environ.* **143**, 85–96 (2014).

27. M. Finer, L. Villa, H. Vale, A. Ariñez, A. Nicolau, K. Walker, MAAP #129: Amazon Fires 2020 – Recap of Another Intense Fire Year (2020); https://www.maaproject.org/2020/amazon-fires-recap/.

28. L. Giglio, L. Boschetti, D. P. Roy, M. L. Humber, C. O. Justice, The collection 6 MODIS burned area mapping algorithm and product. *Remote Sens. Environ.* **217**, 72–85 (2018).

29. T. T. van Leeuwen, G. R. van der Werf, A. A. Hoffmann, R. G. Detmers, G. Rücker, N. H. F. French, S. Archibald, J. A. Carvalho Jr., G. D. Cook, W. J. de Groot, C. Hély, E. S. Kasischke, S. Kloster, J. L. Mc Carty, M. L. Pettinari, P. Savadogo, E. C. Alvarado, L. Boschetti, S. Manuri, C. P. Meyer, F. Siegert, L. A. Trollope, W. S. W. Trollope, Biomass burning fuel consumption rates: A field measurement database. *Biogeosciences* **11**, 7305–7329 (2014).

30. J. K. Balch, D. C. Nepstad, L. M. Curran, P. M. Brando, O. Portela, P. Guilherme, J. D. Reuning-Scherer, O. de Carvalho Jr., Size, species, and fire behavior predict tree and liana mortality from experimental burns in the Brazilian Amazon. *For. Ecol. Manage.* **261**, 68–77 (2011).

31. M. V. F. Silveira, C. A. Petri, I. S. Broggio, G. O. Chagas, M. S. Macul, C. C. S. S. Leite, E. M. M. Ferrari, C. G. V. Amim, A. L. R. Freitas, A. Z. V. Motta, L. M. E. Carvalho, C. H. L. S. Junior, L. O. Anderson, L. E. O. C. Aragão, Drivers of fire anomalies in the Brazilian Amazon: Lessons learned from the 2019 fire crisis. *Landarzt* **9**, 1–24 (2020).

32. Brasil. Decreto No 9985 (2019); www.planalto.gov.br/ccivil_03/_ato2019-2022/2019/decreto/D9985.htm#textoimpressao [accessed 25 August 2021].

33. R. Fu, L. Yin, W. Li, P. A. Arias, R. E. Dickinson, L. Huang, S. Chakraborty, K. Fernandes, B. Liebmann, R. Fisher, R. B. Myneni, Increased dry-season length over southern Amazonia in recent decades and its implication for future climate projection. *Proc. Natl. Acad. Sci. U.S.A.* **110**, 18110–18115 (2013).

34. J. W. Kaiser, A. Heil, M. O. Andreae, A. Benedetti, N. Chubarova, L. Jones, J. J. Morcrette, M. Razinger, M. G. Schultz, M. Suttie, G. R. van der Werf, Biomass burning emissions estimated with a

global fire assimilation system based on observed fire radiative power. *Biogeosciences* **9**, 527–554 (2012).

35. K. Withey, E. Berenguer, A. F. Palmeira, F. D. B. Espírito-Santo, G. D. Lennox, C. V. J. Silva, L. E. O. C. Aragão, J. Ferreira, F. França, Y. Malhi, L. C. Rossi, J. Barlow, Quantifying immediate carbon emissions from El Niño-mediated wildfires in humid tropical forests. *Philos. Trans. R. Soc. B Biol. Sci.* **373**, 20170312 (2018).

36. W. Schroeder, J. T. Morisette, I. Csiszar, D. Morton, C. O. Justice, Characterizing vegetation fire dynamics in brazil through multisatellite data: Common trends and practical issues. *Earth Interact.* **9**, 13 (2005).

37. S. Veraverbeke, F. Sedano, S. J. Hook, J. T. Randerson, Y. Jin, B. M. Rogers, Mapping the daily progression of large wildland fires using MODIS active fire data. *Int. J. Wildl. Fire* **23**, 655–667 (2014).

38. P. Oliva, W. Schroeder, Assessment of VIIRS 375m active fire detection product for direct burned area mapping. *Remote Sens. Environ.* **160**, 144–155 (2015).

39. R. E. Wolfe, G. Lin, M. Nishihama, K. P. Tewari, J. C. Tilton, A. R. Isaacman, Suomi NPP VIIRS prelaunch and on-orbit geometric calibration and characterization. *J. Geophys. Res. Atmos.* **118**, 11508–11521 (2013).

40. F. Li, X. Zhang, S. Kondragunta, D. P. Roy, Investigation of the fire radiative energy biomass combustion coefficient: A comparison of polar and geostationary satellite retrievals over the conterminous United States. *Eur. J. Vasc. Endovasc. Surg.* **123**, 722–739 (2018).

41. G. Roberts, M. J. Wooster, N. Lauret, J. P. Gastellu-Etchegorry, T. Lynham, D. McRae, Investigating the impact of overlying vegetation canopy structures on fire radiative power (FRP) retrieval through simulation and measurement. *Remote Sens. Environ.* **217**, 158–171 (2018).

42. J. K. Balch, D. C. Nepstad, P. M. Brando, L. M. Curran, O. Portela, O. De Carvalho Jr., P. Lefebvre, Negative fire feedback in a transitional forest of southeastern Amazonia. *Glob. Chang. Biol.* **14**, 2276–2287 (2008).

43. Y. Chen, D. C. Morton, Y. Jin, G. J. Collatz, P. S. Kasibhatla, G. R. van der Werf, R. S. DeFries, J. T. Randerson, Long-term trends and interannual variability of forest, savanna and agricultural fires in South America. *Carbon Manag.* **4**, 617–638 (2013).

44. V. Avitabile, M. Herold, G. B. M. Heuvelink, S. L. Lewis, O. L. Phillips, G. P. Asner, J. Armston, P. S. Ashton, L. Banin, N. Bayol, N. J. Berry, P. Boeckx, B. H. J. Jong, B. DeVries, C. A. J. Girardin, E. Kearsley, J. A. Lindsell, G. Lopez-Gonzalez, R. Lucas, Y. Malhi, A. Morel, E. T. A. Mitchard, L. Nagy, L. Qie, M. J. Quinones, C. M. Ryan, S. J. W. Ferry, T. Sunderland, G. V. Laurin, R. C. Gatti, R. Valentini, H. Verbeeck, A. Wijaya, S. Willcock, An integrated pan-tropical biomass map using multiple reference datasets. *Glob. Chang. Biol.* **22**, 1406–1420 (2016).

45. S. S. Saatchi, N. L. Harris, S. Brown, M. Lefsky, E. T. A. Mitchard, W. Salas, B. R. Zutta, W. Buermann, S. L. Lewis, S. Hagen, S. Petrova, L. White, M. Silman, A. Morel, Benchmark map of forest carbon stocks in tropical regions across three continents. *Proc. Natl. Acad. Sci. U.S.A.* **108**, 9899–9904 (2011).

46. A. Baccini, S. J. Goetz, W. S. Walker, N. T. Laporte, M. Sun, D. Sulla-Menashe, J. Hackler, P. S. A. Beck, R. Dubayah, M. A. Friedl, S. Samanta, R. A. Houghton, Estimated carbon dioxide emissions from tropical deforestation improved by carbon-density maps. *Nat. Clim. Chang.* **2**, 182–185 (2012).

47. E. Roteta, A. Bastarrika, M. Padilla, T. Storm, E. Chuvieco, Development of a Sentinel-2 burned area algorithm: Generation of a small fire database for sub-Saharan Africa. *Remote Sens. Environ.* **222**, 1–17 (2019).

48. Mapbiomas, Projeto MapBiomas – Mapeamento das áreas queimadas no Brasil (Coleção 1) (2021) [accessed 20 January 2021]; https://github.com/mapbiomas-brazil/user-toolkit.

49. C. Ichoku, L. Ellison, Global top-down smoke-aerosol emissions estimation using satellite fire radiative power measurements. *Atmos. Chem. Phys.* **14**, 6643–6667 (2014).

50. R. Koster, A. S. Darmenov, A. Silva, The quick fire emissions dataset (QFED): Documentation of versions 2.1, 2.2 and 2.4, NASA technical report series on global modeling and data assimilation. NASA/TM-2015-104606 **38**, (2015).

51. P. H. Freeborn, M. J. Wooster, G. Roberts, Addressing the spatiotemporal sampling design of MODIS to provide estimates of the fire radiative energy emitted from Africa. *Remote Sens. Environ.* **115**, 475–489 (2011).

52. N. Andela, J. W. Kaiser, G. R. Van Der Werf, M. J. Wooster, New fire diurnal cycle characterizations to improve fire radiative energy assessments made from MODIS observations. *Atmos. Chem. Phys.* **15**, 8831–8846 (2015).

53. D. E. Ward, R. A. Susott, J. B. Kauffman, R. E. Babbitt, D. L. Cummings, B. Dias, B. N. Holben, Y. J. Kaufman, R. A. Rasmussen, A. W. Setzer, Smoke and fire characteristics for cerrado and deforestation burns in Brazil: BASE-B experiment. *J. Geophys. Res.* **97**, 14601–14619 (1992).

54. E. A. De Castro, J. B. Kauffman, Ecosystem structure in the Brazilian Cerrado: A vegetation gradient of aboveground biomass, root mass and consumption by fire. *J. Trop. Ecol.* **14**, 263–283 (1998).

55. G. J. Huffman, D. T. Bolvin, E. J. Nelkin, J. Tan, Integrated multi-satellitE retrievals from GPM (IMERG), version 6. NASA's precipitation processing center (2014) [accessed 7 May 2020]; ftp://arthurhouftps.pps.eosdis.nasa.gov/.

56. S. Nunes, L. Oliveira Jr., J. Siqueira, D. C. Morton, C. M. Souza Jr., Unmasking secondary vegetation dynamics in the Brazilian Amazon. *Environ. Res. Lett.* **15**, 034057 (2020).
